# Development and validation of a clinical model for preconception and early pregnancy risk prediction of gestational diabetes mellitus in nulliparous women

**DOI:** 10.1371/journal.pone.0215173

**Published:** 2019-04-12

**Authors:** Brittney M. Donovan, Patrick J. Breheny, Jennifer G. Robinson, Rebecca J. Baer, Audrey F. Saftlas, Wei Bao, Andrea L. Greiner, Knute D. Carter, Scott P. Oltman, Larry Rand, Laura L. Jelliffe-Pawlowski, Kelli K. Ryckman

**Affiliations:** 1 Department of Epidemiology, University of Iowa College of Public Health, Iowa City, Iowa, United States of America; 2 Department of Biostatistics, University of Iowa College of Public Health, Iowa City, Iowa, United States of America; 3 Department of Pediatrics, University of California San Diego, La Jolla, California, United States of America; 4 California Preterm Birth Initiative, University of California San Francisco, San Francisco, California, United States of America; 5 Department of Obstetrics and Gynecology, University of Iowa Carver College of Medicine, Iowa City, Iowa, United States of America; 6 Department of Epidemiology and Biostatistics, University of California San Francisco, San Francisco, California, United States of America; 7 Department of Obstetrics, Gynecology & Reproductive Sciences, University of California San Francisco, San Francisco, California, United States of America; 8 Department of Pediatrics, University of Iowa Carver College of Medicine, Iowa City, Iowa, United States of America; Helmholtz Zentrum München, GERMANY

## Abstract

Implementation of dietary and lifestyle interventions prior to and early in pregnancy in high risk women has been shown to reduce the risk of gestational diabetes mellitus (GDM) development later in pregnancy. Although numerous risk factors for GDM have been identified, the ability to accurately identify women before or early in pregnancy who could benefit most from these interventions remains limited. As nulliparous women are an under-screened population with risk profiles that differ from their multiparous counterparts, development of a prediction model tailored to nulliparous women may facilitate timely preventive intervention and improve maternal and infant outcomes. We aimed to develop and validate a model for preconception and early pregnancy prediction of gestational diabetes mellitus based on clinical risk factors for nulliparous women. A risk prediction model was built within a large California birth cohort including singleton live birth records from 2007–2012. Model accuracy was assessed both internally and externally, within a cohort of women who delivered at University of Iowa Hospitals and Clinics between 2009–2017, using discrimination and calibration. Differences in predictive accuracy of the model were assessed within specific racial/ethnic groups. The prediction model included five risk factors: race/ethnicity, age at delivery, pre-pregnancy body mass index, family history of diabetes, and pre-existing hypertension. The area under the curve (AUC) for the California internal validation cohort was 0.732 (95% confidence interval (CI) 0.728, 0.735), and 0.710 (95% CI 0.672, 0.749) for the Iowa external validation cohort. The model performed particularly well in Hispanic (AUC 0.739) and Black women (AUC 0.719). Our findings suggest that estimation of a woman’s risk for GDM through model-based incorporation of risk factors accurately identifies those at high risk (i.e., predicted risk >6%) who could benefit from preventive intervention encouraging prompt incorporation of this tool into preconception and prenatal care.

## Introduction

Gestational diabetes mellitus (GDM) is the most common metabolic complication in pregnancy affecting 7% of pregnancies globally.[1,2] Women who develop GDM experience higher rates of hypertension and pre-eclampsia during pregnancy and are more likely to need intervention during labor and delivery by way of assisted vaginal delivery, induction of labor, and cesarean delivery for dystocia and fetal distress.[3] Intra-uterine exposure to metabolic alterations and epigenetic programming can lead to excessive growth of the fetus along with a myriad of medical complications after delivery, including infant respiratory distress syndrome, cardiomyopathy, and hypoglycemia.[4–6] Later in life, women and their offspring face an increased risk of developing metabolic syndrome, obesity, type 2 diabetes, and cardiovascular disease.[3,4,7] Due to increasing prevalence and the heightened risk of adverse outcomes as a consequence of exposure to hyperglycemia during pregnancy, universal glucose screening for GDM at 24–28 weeks gestation has become the standard practice in most developed nations.[8,9] However, there is accumulating evidence to suggest that implementing lifestyle interventions prior to or earlier in pregnancy (before the 20th week of gestation) in high risk women could limit gestational weight gain and reduce the risk of developing GDM later in pregnancy.[10–12]

Although numerous risk factors for GDM have been identified, the ability to accurately identify women before or early in pregnancy who are at risk for developing GDM remains limited. Previously developed models for preconception and early pregnancy prediction of GDM have likely not been implemented in clinical care due to insufficient external validation and evaluation of clinical utility.[13,14] Additionally, these models primarily rely on previous history of GDM as the strongest predictor of subsequent GDM,[15] which is not applicable in nulliparous women (i.e., women who have never carried a pregnancy to 20 weeks gestation or more). Nulliparous women are an under-screened population with risk profiles that differ from their multiparous counterparts.[16,17] Development of a prediction model tailored to nulliparous women may increase the ability to identify those at high risk for GDM to facilitate timely preventive intervention and improve maternal and infant outcomes.

The objective of this study was to rigorously develop and validate a clinical model for preconception and early pregnancy prediction of GDM risk based on clinical risk factors for nulliparous women. As the prevalence and importance of certain risk factors for GDM have been shown to vary across racial/ethnic groups,[18] we also performed stratified modeling to assess for improved prediction among nulliparous women in specific racial/ethnic groups. To our knowledge, this is the first study to develop and validate a model for preconception and early GDM risk factor screening in nulliparous women and assess model performance within specific racial/ethnic groups, which is important for generalizability.

## Methods

### Study populations and data collection

#### California birth cohort

**Fig 1:**
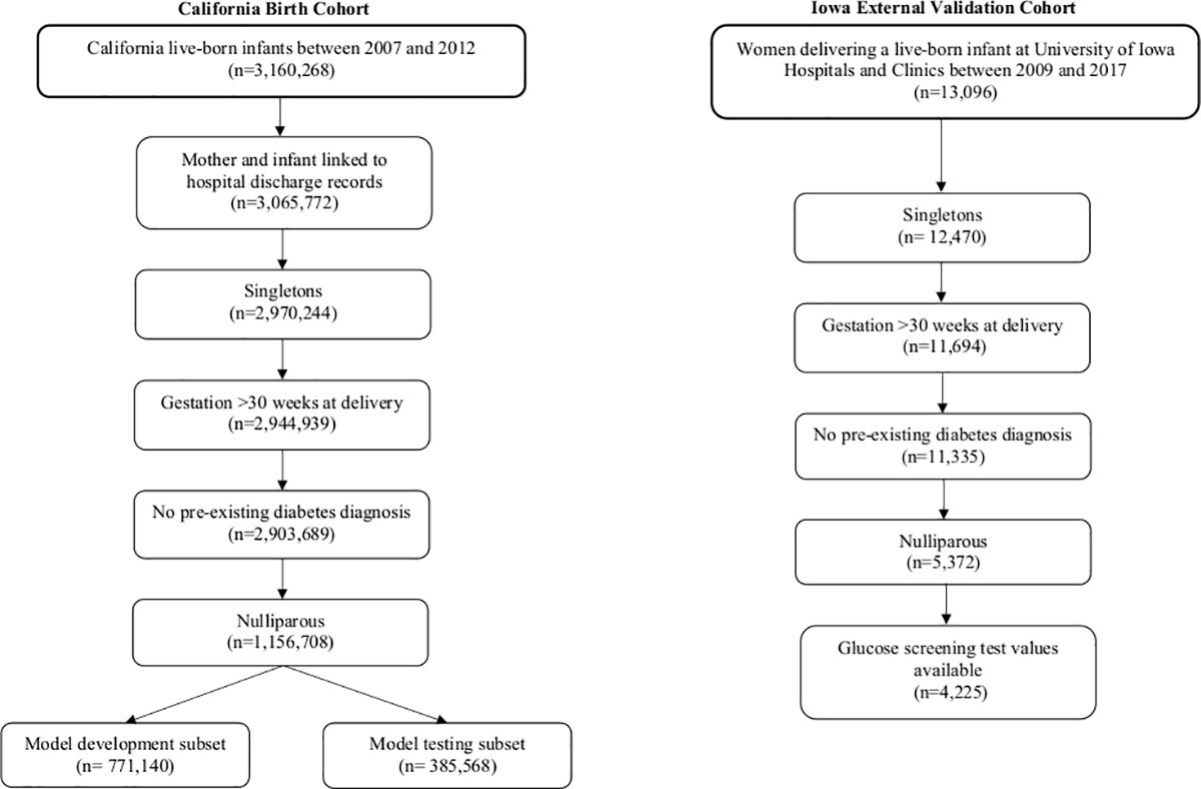
Sample selection in the California and Iowa cohorts.

The study population used for model development and internal validation was drawn from singleton live births in California from 2007–2012 in a birth cohort file maintained by the California Office of Statewide Health Planning and Development. This database includes linked infant vital statistics (birth and death certificate data) and mother and infant hospital discharge records for the nine months prior to delivery and one year post-delivery. Linkage was performed by California Office of Statewide Health Planning and Development analytical personnel through comparison of date of birth, birth weight, and birth time across records; de-identified data was then provided for analyses. We leveraged the existing records of nulliparous women who delivered a singleton live birth during the study period and had linked hospital discharge records. Women with pre-existing type 1 or type 2 diabetes mellitus were excluded. Those delivering at <30 weeks gestation were also excluded to ensure that all women included in this cohort had the opportunity to be screened for and potentially diagnosed with GDM. Parity was ascertained from birth certificate records. Gestational age at delivery was determined using the birth certificate reported ‘best obstetric estimate’, which is based on last menstrual period and ultrasound dating, when available.[19] Our final cohort included 1,156,708 nulliparous women (Fig 1).

GDM was identified from maternal diagnosis of *International Classification of Diseases*, 9th Revision, Clinical Modification (ICD-9-CM) code 648.8 for ‘pregnancy complicated by abnormal glucose tolerance’ or birth certificate indication of diagnosis of diabetes during pregnancy. Pre-existing type 1 and type 2 diabetes used for study exclusion were identified using ICD-9-CM codes 648.0 (‘diabetes mellitus complicating pregnancy’) and 250 (‘diabetes mellitus’) or birth certificate indication of pre-pregnancy diabetes diagnosis.

Information was collected on maternal factors known to be associated with GDM development. Maternal age at delivery, race and ethnicity, pre-pregnancy body mass index (BMI), and education level were ascertained using birth certificate records. Information on smoking status during pregnancy (ICD-9-CM code 649.0 for ‘tobacco use disorder complicating pregnancy, childbirth, or the puerperium’) and artificial reproductive technology (ICD-9-CM code V23.85 for ‘pregnancy resulting from assisted reproductive technology’) was collected from hospital discharge or birth certificate records. Information on expected payer for delivery, family history of diabetes (ICD-9-CM code V18.0 for ‘family history of diabetes mellitus’), polycystic ovarian syndrome (PCOS) (ICD-9-CM code 256.4 for ‘polycystic ovaries’), pre-existing hypertension (ICD-9-CM codes 642.0, 642.1, 642.2, and 642.7 for ‘benign essential hypertension complicating pregnancy, childbirth, and the puerperium’, ‘hypertension secondary to renal disease complicating pregnancy, childbirth, and the puerperium’, ‘other pre-existing hypertension complicating pregnancy, childbirth, and the puerperium’, and ‘pre-eclampsia or eclampsia superimposed on pre-existing hypertension’), pre-existing dyslipidemia (ICD-9-CM code 272 for ‘disorders of lipoid metabolism’), personal history of cardiovascular disease (CVD) (ICD-9-CM code V12.5 for ‘personal history of diseases of circulatory system’), and personal history of miscarriage (i.e., a non-viable pregnancy with delivery before 20 weeks gestation) (ICD-9-CM code V23.2 for ‘supervision of high risk pregnancy with history of abortion’) were ascertained from hospital discharge files. Women with missing predictor data were excluded from further analyses.

#### Iowa external validation cohort

All women delivering a live-born infant at the University of Iowa Hospitals and Clinics (UIHC) from 2009–2017 were eligible for inclusion in the Iowa external validation cohort (Fig 1). Through medical chart review, 5,372 nulliparous women were identified after exclusion of multiple births, women delivering at <30 weeks gestation, and those with a pre-existing type 1 or type 2 diabetes diagnosis.

GDM status was ascertained through assessment of glucose screening test values. Blood glucose was measured at UIHC and recorded in patient medical records. In accordance with UIHC guidelines, women were diagnosed with GDM using the two-step approach for universal glucose testing.[20] Women missing predictor data or glucose screening test values were excluded from further analyses.

Demographic variables, including maternal age at delivery, race and ethnicity, education level, and expected payer for delivery, were coded to mimic variables within the California birth cohort file (i.e., categorical variables were grouped similarly). Pre-pregnancy BMI (kg/m^2^) was calculated from height and weight recorded during the clinic visit closest to the estimated conception date. Individuals with a recorded smoking history listed as ‘current smoker’ between the estimated conception date (calculated by subtracting the gestational age in days from the delivery date) and delivery dates were defined as having smoked during pregnancy. The same ICD-9-CM codes, as identified within the California birth cohort file, were used to determine family history of diabetes, PCOS diagnosis, pre-existing hypertension, pre-existing dyslipidemia, personal history of CVD, assisted reproductive technology use, and personal history of miscarriage within the Iowa cohort.

### Statistical analysis

#### Model development

**Fig 2:**
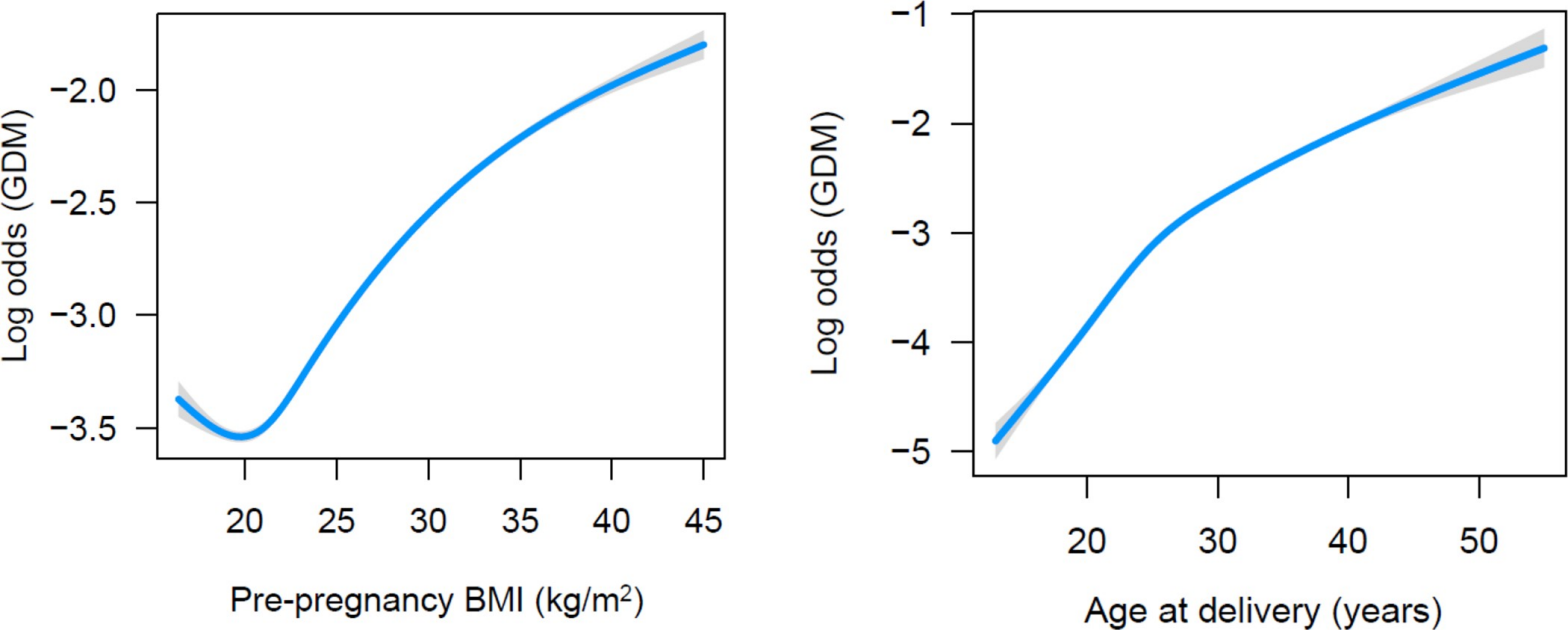
Associations between pre-pregnancy body mass index (BMI) and age at delivery and gestational diabetes mellitus.

The California cohort was randomly divided into a development (2/3 of total: n = 771,140) and a testing (1/3 of total: n = 385,568) subset. The prediction model was built within the model development subset. Maternal demographic and clinical characteristics were compared using univariate and multivariable logistic regression between women who were diagnosed with GDM and those who were not. All identified maternal demographic and clinical characteristics (i.e., race/ethnicity, age at delivery, education, expected payer for delivery, smoked during pregnancy, pre-pregnancy BMI, family history of diabetes, PCOS diagnosis, pre-existing hypertension, pre-existing dyslipidemia, personal history of CVD, assisted reproductive technology use, and personal history of miscarriage) were initially included in the multivariate logistic regression model. To address non-linear relationships between pre-pregnancy BMI and age at delivery and the log odds of GDM (Fig 2), a natural cubic spline was fit for these variables with 5 degrees of freedom. Multicollinearity between predictor variables was assessed using condition indices and variance proportions.[21] Education was found to be multicollinear with age at delivery and expected payer for delivery and was initially removed from the model. Non-significant variables (two-sided *P* >0.001) were subsequently removed from the model. A more stringent alpha level (two-sided *P* <0.001) was used for assessing significant variables due to the large sample size which enabled smaller effect sizes to be detected. To determine the importance of the remaining variables, variables were sequentially removed based on their χ^2^ statistic (with lower values being selected for removal first).[22] A variable was considered significant and kept in the final prediction model if, upon removal, the model discrimination (area under the curve (AUC)) was reduced or if the AUC remained unchanged but the beta coefficients for the remaining variables changed by >15% from the full model.[23,24]

Prevalence and importance of certain risk factors for GDM have been shown to vary across racial/ethnic groups.[18] Therefore, stratified modeling was also undertaken to assess for improved prediction among nulliparous women of certain racial/ethnic groups. Prediction models were built within each racial/ethnic group using the same variables and selection procedure as described for the entire nulliparous cohort. A natural cubic spline was fit for pre-pregnancy BMI and age at delivery with 5 degrees of freedom within each model to meet regression assumptions. Education was found to be multicollinear with age at delivery and expected payer for delivery among all racial/ethnic groups and was initially removed from the models. Pairwise interactions were assessed for each variable remaining in the models. Significant interactions were further examined using predicted probability plots.

To estimate a woman’s risk of developing GDM, predicted risk was calculated from model coefficients using the formula: 1/ (1 + e^-(*t*)^), where *t* is the combination of explanatory variables derived from the logistic regression analysis.[22,25]

#### Model testing and validation

The final risk prediction model was tested within the California model testing subset and the Iowa cohort to assess both internal and external validity. Model accuracy was assessed using discrimination and calibration. Discrimination was assessed using receiver operating characteristic curves and corresponding AUCs. Calibration, related to goodness of fit, was assessed in calibration plots by comparing the predicted risk of GDM for each woman to her observed outcome. Predicted risks for 10 groups of equal size were plotted on the x-axis and the mean observed outcome was plotted on the y-axis, with the 45° line indicative of perfect calibration.[26] Overestimation and underestimation of the predictions as well as overfitting of the model were assessed by examining the calibration intercept and slope of the linear prediction. A calibration intercept <0 indicates overestimation (i.e., the model predictions are too high), an intercept >0 indicates underestimation, and a calibration slope <1 indicates overfitting.[13,27] Testing and validation of the stratified models was assessed in a manner similar to that of the primary analysis.

#### Risk stratification

Disease management and screening programs often use threshold-based models to select individuals at highest risk for disease, as they would benefit most from intervention strategies.[28] To classify individuals into high risk and low risk categories, individual predicted risks were converted into binary categories using a chosen threshold. The threshold was determined based on the smallest distance from the receiver operating characteristic curve to the perfect classification point (i.e., upper left corner of the receiver operating characteristic space).[29] Both the sensitivity and specificity were maximized at this point, which is desirable for screening tests.[30] Using this threshold, individuals in the California model testing subset and the Iowa cohort were divided into two GDM risk groups: 1) women below the threshold (low risk) and 2) women above the threshold (high risk). Sensitivity, specificity, positive predictive value, negative predictive value, and correct classification rates were computed from the risk prediction model at the specified threshold. A secondary analysis was performed to assess the performance of the final model at various predicted risk thresholds.

Analyses were conducted using SAS version 9.4 (SAS Institute Inc., Cary, NC, USA) and R-software version 3.5.1 (R foundation for statistical computing, Vienna, Austria. URL http://www.R-project.org). Analysis code is available at https://osf.io/w7aes/. Methods and protocols for this study were approved by the Committee for the Protection of Human Subjects within the Health and Human Services Agency of the State of California. California birth cohort data used in this analysis is owned by the State of California who grants access through an application and approval process. This process is open to any interested researcher or other investigator who seeks access. No special permission was granted for this project. Interested researchers may apply for access to the data at https://oshpd.ca.gov/data-and-reports/request-data/for-researchers/ or directly contact dataandreports@oshpd.ca.gov. Data provided to the researchers by California Office of Statewide Health Planning and Development was de-identified and determined not to qualify as human subjects research by the University of Iowa Institutional Review Board (IRB no.: 201602793). Data collected from the University of Iowa Hospitals and Clinics contains sensitive patient information and ethical restrictions on sharing this information is managed by the University of Iowa Institutional Review Board. The University of Iowa Institutional Review Board granted a waiver of informed consent for retrospective data analysis from patients who received services at the University of Iowa Hospitals and Clinics (IRB no.: 201706737). Results are reported in compliance with the Transparent Reporting of a multivariable prediction model for Individual Prognosis or Diagnosis (TRIPOD) criteria (S1 File).[31]

## Results

### Cohort characteristics

Demographic and clinical characteristics of the study cohorts are shown in Table 1. 6.3% of California women were diagnosed with GDM, while 4.3% of Iowa women had glucose-confirmed GDM. Characteristics of the California and Iowa cohorts differed substantially. Hispanic ethnicity made up over 40% of the nulliparous population within the California birth cohort, while most women within the Iowa cohort were non-Hispanic White (73.7%). Women within the California cohort were younger, less educated, more likely to be on government insurance, and had lower BMIs and smoking rates during pregnancy than women within the Iowa cohort.

**Table 1:** Demographic and clinical characteristics of nulliparous women within the California, 2007–2012, and Iowa, 2009–2017, study cohorts.

	California Cohort			
	Total Nulliparous Population n (%)	Model Development Subset n (%)	Model Testing Subset n (%)	Iowa Cohort n (%)
**Total sample size**	**1,156,708**	**771,140**	**385,568**	**4,225**
**GDM diagnosis**	73,017 (6.3)	48,608 (6.3)	24,409 (6.3)	181 (4.3)
**Race/ethnicity**				
White, not Hispanic	342,521 (29.6)	228,219 (29.6)	114,302 (29.7)	3,114 (73.7)
Hispanic	492,520 (42.6)	328,517 (42.6)	164,003 (42.5)	248 (5.9)
Black	63,205 (5.5)	42,041 (5.5)	21,164 (5.5)	325 (7.7)
Asian	167,993 (14.5)	111,698 (14.5)	56,295 (14.6)	381 (9.0)
AI/AN	4,922 (0.4)	3,295 (0.4)	1,627 (0.4)	—
H/PI	4,456 (0.4)	2,959 (0.4)	1,497 (0.4)	—
Other racial group^†^	81,091 (7.0)	54,411 (7.1)	26,680 (6.9)	140 (3.3)
**Age at delivery (years)**				
Mean (SD)	25.9 (6.3)	25.9 (6.3)	25.9 (6.3)	27.8 (5.3)
Median (range)^ǂ^	25.0 (13.0–55.0)	25.0 (13.0–55.0)	25.0 (13.0–55.0)	28.0 (24.0–31.0)
Missing	59	50	9	0
**Education**				
<12 years	210,881 (18.9)	140,968 (19.0)	69,913 (18.8)	195 (5.8)
12 years	288,315 (25.9)	192,287 (25.9)	96,028 (25.9)	444 (13.1)
>12 years	614,301 (55.2)	409,001 (55.1)	205,300 (55.3)	2,745 (81.1)
Missing	43,211	28,884	14,327	841
**Expected payer for delivery**				
Government	522,817 (45.2)	348,893 (45.2)	173,924 (45.1)	909 (23.8)
Private	590,973 (51.1)	393,662 (51.1)	197,311 (51.2)	2,822 (73.9)
Other	42,890 (3.7)	28,568 (3.7)	14,322 (3.7)	90 (2.4)
Missing	28	17	11	404
**Smoked during pregnancy**	47,312 (4.1)	31,590 (4.1)	15,722 (4.1)	441 (10.4)
**Pre-pregnancy BMI (kg/m**^**2**^**)**				
Mean (SD)	24.6 (5.1)	24.6 (5.1)	24.6 (5.1)	27.3 (6.6)
Median (range)^ǂ^	23.4 (16.4–45.0)	23.4 (16.4–45.0)	23.4 (16.4–45.0)	25.5 (22.8–30.1)
Missing	96,990	64,431	32,559	80
**Family history of diabetes**	9,914 (0.9)	6,623 (0.9)	3,291 (0.9)	38 (0.9)
**PCOS diagnosis**	2,549 (0.2)	1,729 (0.2)	820 (0.2)	199 (4.7)
**Pre-existing hypertension**	13,384 (1.2)	8,990 (1.2)	4,394 (1.1)	—
**Pre-existing dyslipidemia**	2,438 (0.2)	1,624 (0.2)	814 (0.2)	76 (1.8)
**Personal history of CVD**	2,001 (0.2)	1,365 (0.2)	636 (0.2)	28 (0.7)
**Assisted reproductive technology use**	9,248 (0.8)	6,194 (0.8)	3,054 (0.8)	—
**Personal history of miscarriage**	3,652 (0.3)	2,429 (0.3)	1,223 (0.3)	16 (0.4)

GDM, gestational diabetes mellitus; AI/AN, American Indian/Alaska Native; H/PI, Hawaiian/Pacific Islander; SD, standard deviation; BMI, body mass index; PCOS, polycystic ovarian syndrome; CVD, cardiovascular disease.

^†^Includes two or more races and race unknown.

^ǂ^Range = minimum value- maximum value.

—Data suppressed (n <10).

### Model development

Missing variable information for women included in the California model development subset is outlined in S1 Table. 8.4% of women were excluded (n = 64,466) due to missing predictor data. Women excluded were more likely to be Hispanic or Black and have pre-existing hypertension than included women.

Maternal characteristics are compared between women who were diagnosed with GDM and those who were not in S2 Table. From the thirteen variables evaluated in the California model development subset, five variables (race/ethnicity, age at delivery, pre-pregnancy BMI, family history of diabetes, and pre-existing hypertension) were retained in the final model. Model estimates for the variables within the final model are shown in S3 Table. Increased age at delivery, higher pre-pregnancy BMI, and Asian race were found to be the strongest risk factors.

### Model testing and validation

The final model was tested within the California model testing subset and the Iowa cohort (see S4 Table for characteristics of women with and without GDM in the California model testing subset and Iowa cohort). Missing variable information for women included in both validation cohorts is outlined in S5 Table. 8.4% of women (n = 32,565) in the California model testing subset and 1.9% (n = 80) of women in the Iowa cohort were excluded due to missing predictor data. Women excluded from the California model testing subset had lower pre-pregnancy BMIs and were more likely to be Hispanic or Black and have pre-existing hypertension than included women. Women excluded from the Iowa cohort were younger at delivery and were more likely to be Black than those who were included.

Glucose screening test data were available for outcome ascertainment for 4,225 (78.6%) nulliparous women within the Iowa cohort (Fig 1). The characteristics of women excluded for not having glucose screening test values were marginally different than those among the included women (S6 Table). A higher proportion of women with glucose values were Asian and had private insurance than those without values. Included women also had slightly lower pre-pregnancy BMIs (27.3 kg/m^2^ vs. 31.0 kg/m^2^), were less likely to smoke during pregnancy, and were slightly older (27.8 years vs. 25.7 years) than women without glucose screening test values.

**Fig 3:**
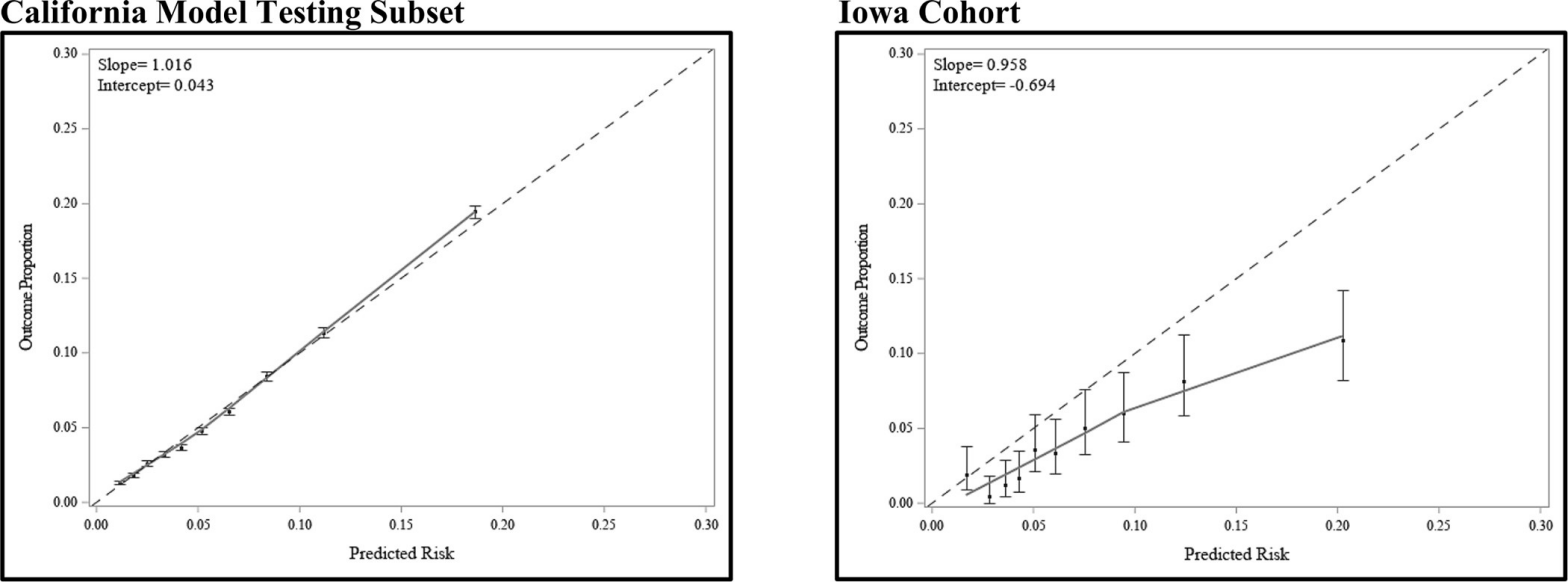
Calibration plots of the final model internally validated within the California model testing subset and externally validated within the Iowa cohort. The final model was built using the entire nulliparous cohort within the California model development subset and includes the following variables: race/ethnicity, age at delivery (natural cubic spline transformed), pre-pregnancy BMI (natural cubic spline transformed), family history of diabetes, and pre-existing hypertension. The dotted diagonal line indicates perfect calibration (intercept = 0 and slope = 1). Dots represent the observed proportion of events by the predicted risk for 10 groups of equal size, with vertical lines representing 95% confidence intervals.

Performance measures for the risk prediction model built using the entire nulliparous cohort in the California model development subset and tested within the California model testing subset and Iowa cohort are presented in Table 2. The final model showed moderate capacity to discriminate between women with and without GDM in the California model testing subset (AUC 0.732 (95% confidence interval (CI): 0.728, 0.735)). Model discrimination was slightly reduced when externally validating the model within the Iowa cohort (AUC 0.710 (95% CI: 0.672, 0.749)). The calibration plot intercept and slope indicate that the model was well calibrated within the California model testing subset (Fig 3). The final model tended to overestimate the risk of GDM in the Iowa cohort, particularly for women at the highest risk level as the predicted risks were higher than the observed risks.

**Table 2:** Accuracy of the final and stratified models among all nulliparous women and nulliparous women of specific racial/ethnic groups in the California model testing subset and Iowa cohort.

Racial/Ethnic Group	California Model Testing Subset				Iowa Cohort			
	Number of subjects included in model	AUC (95% CI)	Calibration Plot Statistics		Number of subjects included in model	AUC (95% CI)	Calibration Plot Statistics	
			Intercept	Slope			Intercept	Slope
All Nulliparous Women*	353,003	0.732 (0.728, 0.735)	0.043	1.016	4,145	0.710 (0.672, 0.749)	-0.694	0.958
**White, not Hispanic Women**								
Model built within all nulliparous women*	106,808	0.693 (0.685, 0.700)	-0.018	0.989	3,063	0.723 (0.676, 0.769)	-0.452	1.081
Model built within this group^†^	106,808	0.693 (0.685, 0.700)	-0.034	0.983	3,063	0.713 (0.666, 0.760)	-0.841	0.946
**Hispanic Women**								
Model built within all nulliparous women*	148,703	0.739 (0.733, 0.745)	0.085	1.034	—	—	—	—
Model built within this group^‡^	148,703	0.740 (0.735, 0.746)	0.068	1.027	—	—	—	—
**Black Women**								
Model built within all nulliparous women*	18,853	0.719 (0.700, 0.738)	-0.473	0.867	—	—	—	—
Model built within this group ^¥^	18,853	0.718 (0.699, 0.737)	-0.488	0.861	—	—	—	—
**Asian Women**								
Model built within all nulliparous women*	51,246	0.665 (0.658, 0.672)	0.134	1.060	—	—	—	—
Model built within this group ^€^	51,242	0.666 (0.659, 0.673)	0.146	1.066	—	—	—	—
**Hawaiian/Pacific Islander Women**								
Model built within all nulliparous women*	1,328	0.715 (0.666, 0.764)	-0.009	1.028	—	—	—	—
Model built within this group ^§^	1,328	0.709 (0.660, 0.757)	-0.117	0.978	—	—	—	—
**Women in Other Racial Groups**								
Model built within all nulliparous women*	24,610	0.713 (0.700, 0.727)	-0.089	0.969	—	—	—	—
Model built within this group ^¢^	24,607	0.715 (0.701, 0.728)	-0.072	0.975	—	—	—	—

AUC, area under the curve; CI, confidence interval.

* Final model included the following variables: race/ethnicity, age at delivery (natural cubic spline transformed), pre-pregnancy body mass index (natural cubic spline transformed), family history of diabetes, and pre-existing hypertension.

^†^ Model included the following variables: age at delivery (natural cubic spline transformed), pre-pregnancy body mass index (natural cubic spline transformed), family history of diabetes, polycystic ovarian syndrome diagnosis, pre-existing hypertension, age at delivery (natural cubic spline transformed) x pre-pregnancy body mass index (natural cubic spline transformed), and pre-pregnancy body mass index (natural cubic spline transformed) x pre-existing hypertension.

^‡^ Model includes the following variables: age at delivery (natural cubic spline transformed), pre-pregnancy body mass index (natural cubic spline transformed), expected payer for delivery, family history of diabetes, pre-existing hypertension, and age at delivery (natural cubic spline transformed) x expected payer for delivery.

^¥^ Model included the following variables: age at delivery (natural cubic spline transformed), pre-pregnancy body mass index (natural cubic spline transformed), and pre-existing hypertension.

^€^ Model included the following variables: age at delivery (natural cubic spline transformed), pre-pregnancy body mass index (natural cubic spline transformed), expected payer for delivery, family history of diabetes, and age at delivery (natural cubic spline transformed) x expected payer for delivery.

^§^ Model included the following variables: age at delivery (natural cubic spline transformed) and pre-pregnancy body mass index (natural cubic spline transformed).

^¢^ Model included the following variables: age at delivery (natural cubic spline transformed), pre-pregnancy body mass index (natural cubic spline transformed), expected payer for delivery, family history of diabetes, pre-existing hypertension, and age at delivery (natural cubic spline transformed) x expected payer for delivery.

—, Model validity was questionable due to the limited number of subjects within this racial/ethnic group with the outcome.

The final model built using the entire nulliparous cohort performed equally as well in each racial/ethnic group as the model built within each group separately (when assessed in both the California model testing subset and Iowa cohort), indicating that the race-specific models were not necessary (Table 2). The final model and stratified models were not tested within the American Indian/Alaska Native group in the California model testing subset and any racial/ethnic group other than White, not Hispanic in the Iowa cohort as these groups did not contain at least 100 subjects with the outcome of interest (S7–S13 Tables).[31] Discrimination and calibration for the final model varied widely across the racial/ethnic groups in the California model testing subset. The model showed the strongest predictive performance among Hispanic women (AUC 0.739 (95% CI: 0.733, 0.745)), with the weakest predictive performance observed among Asian women (AUC (0.665 (95% CI: 0.658, 0.672)). Calibration intercepts and slopes were close to 0 and 1, in most racial/ethnic groups, indicating good model fit. The model slightly overestimated the risk of GDM in Black women (calibration intercept: -0.473).

The parsimonious nature of the final risk prediction model did not lead to a reduction in the accuracy of predicting GDM, justifying its use over a more complex model (S14 Table). A robust model including a limited number of predictors will be easier to implement for targeting GDM preventive interventions. Box 1 outlines the formula for the final model to calculate the individual predicted risk of developing of GDM. Three clinical scenarios were used to demonstrate how this model can be used in clinical practice (S15 Table**).** The predicted risk calculator is available at https://ph-shiny.iowa.uiowa.edu/pbreheny/gdm-risk-calculator.

**Box 1. Model for preconception and early pregnancy prediction of gestational diabetes mellitus risk in nulliparous women**

**FINAL MODEL**

Predicted Risk of Developing Gestational Diabetes Mellitus = [1 / 1 + e^-(*t*)^] where,

*t* = -9.478 + (0.391 x Hispanic) + (0.001 x Black) + (1.064 x Asian) + (0.180 x American Indian/Alaska Native) + (0.644 x Hawaiian/Pacific Islander) + (0.338 x Other racial group) + Age at delivery (natural cubic spline transformed) + Pre-pregnancy body mass index (natural cubic spline transformed) + (0.685 x Family history of diabetes) + (0.533 x Pre-existing hypertension)

### Risk stratification

The performance of the risk stratification strategy is summarized in Table 3. Women’s predicted risk of developing GDM ranged from 0.3%-73.1% (mean 6.3%, standard deviation 5.4%) in the California model testing subset and 0.5%- 46.3% (mean 7.3%, standard deviation 5.6%) in the Iowa cohort. Using the final prediction model and giving equal weight to false positives and false negatives, the optimal threshold for predicted risk was determined to be 6% among all nulliparous women within the California model development subset. Using this threshold, 38.3% of women within the California model testing subset and 45.1% of women within the Iowa cohort were considered high risk for GDM. The risk prediction model had moderate sensitivity and specificity within the California model testing subset (70.8 (95% CI: 70.2, 71.4) and 63.9 (95% CI: 63.7, 64.0)) and the Iowa cohort (76.7 (95% CI: 70.5, 83.0) and 56.3 (95% CI: 54.7, 57.8)), and correctly classified around 60% of GDM cases and non-cases in both cohorts. The positive predictive value was low (11.6% for the California model testing subset and 7.2% for the Iowa cohort) and the negative predictive value was high (97.0% for the California model testing subset and 98.2% for the Iowa cohort).

**Table 3:** Performance of the risk stratification strategy within the California model testing subset and Iowa cohort.

Model Application^†^	Validation Cohort	Number of subjects	True GDM Prevalence n (%)	Women above threshold (‘high risk’) n (%)	Sensitivity (95% CI)	Specificity (95% CI)	PPV (95% CI)	NPV (95% CI)	Correctly Classified n (%)
All Nulliparous Women	California Model Testing Subset	353,003	22,194 (6.3)	135,262 (38.3)	70.8 (70.2, 71.4)	63.9 (63.7, 64.0)	11.6 (11.4, 11.8)	97.0 (97.0, 97.1)	226,967 (64.3)
	Iowa Cohort	4,145	176 (4.3)	1,871 (45.1)	76.7 (70.5, 83.0)	56.3 (54.7, 57.8)	7.2 (6.0, 8.4)	98.2 (97.7, 98.7)	2,368 (57.1)
White, not Hispanic Women	California Model Testing Subset	106,808	5,549 (5.2)	29,181 (27.3)	54.6 (53.3, 55.9)	74.2 (73.9, 74.4)	10.4 (10.0, 10.7)	96.8 (96.6, 96.9)	78,134 (73.2)
	Iowa Cohort	3,063	115 (3.8)	1,224 (40.0)	73.9 (65.9, 81.9)	61.4 (59.6, 63.1)	6.9 (5.5, 8.4)	98.4 (97.8, 99.0)	1,894 (61.8)
Hispanic Women	California Model Testing Subset	148,703	8,085 (5.4)	46,651 (31.4)	65.0 (64.0, 66.1)	70.6 (70.3, 70.8)	11.3 (11.0, 11.6)	97.2 (97.1, 97.3)	104,481 (70.3)
	Iowa Cohort	—	—	—	—	—	—	—	—
Black Women	California Model Testing Subset	18,853	720 (3.8)	3,945 (20.9)	49.3 (45.7, 53.0)	80.2 (79.6, 80.8)	9.0 (8.1, 9.9)	97.6 (97.3, 97.8)	14,898 (79.0)
	Iowa Cohort	—	—	—	—	—	—	—	—
Asian Women	California Model Testing Subset	51,246	6,155 (12.0)	45,100 (88.0)	96.3 (95.8, 96.8)	13.1 (12.8, 13.4)	13.1 (12.8, 13.5)	96.3 (95.8, 96.8)	11,847 (23.1)
	Iowa Cohort	—	—	—	—	—	—	—	—
H/PI Women	California Model Testing Subset	1,328	114 (8.6)	775 (58.4)	80.7 (73.5, 88.0)	43.7 (41.0, 46.5)	11.9 (9.6, 14.2)	96.0 (94.4, 97.7)	623 (46.9)
	Iowa Cohort	—	—	—	—	—	—	—	—
Women in Other Racial Groups	California Model Testing Subset	24,610	1,477 (6.0)	9,253 (37.6)	67.3 (64.9, 69.7)	64.3 (63.7, 64.9)	10.7 (10.1, 11.4)	96.9 (96.6, 97.1)	15,868 (64.5)
	Iowa Cohort	—	—	—	—	—	—	—	—

GDM, gestational diabetes mellitus; CI, confidence interval; PPV, positive predictive value; NPV, negative predictive value AI/AN, American Indian/Alaska Native; H/PI, Hawaiian/Pacific Islander.

^†^Final model included the following variables: race/ethnicity, age at delivery (natural cubic spline transformed), pre-pregnancy BMI (natural cubic spline transformed), family history of diabetes, and pre-existing hypertension.

A 6% predicted risk threshold was applied to determine ‘low’ and ‘high’ risk.

—, Model validity was questionable due to the limited number of participants within this racial/ethnic group with the outcome.

In a secondary analysis, the performance of the final model was assessed at various predicted risk thresholds (S16 Table). While the sensitivity of the risk prediction model increased when the predicted risk threshold was reduced from 6% to 3% (70.8% to 90.1% for the California model testing subset and 76.7% to 94.9% for the Iowa cohort), the specificity and the proportion correctly classified were significantly reduced (63.9% to 32.9% and 64.3% to 36.5% in the California model testing subset and 56.3% to 17.5% and 57.1% to 20.8% in the Iowa cohort) and the proportion of women who were considered high risk was nearly doubled (38.3% to 68.6% for the California model testing subset and 45.1% to 83.0% for the Iowa cohort). When the predicted risk threshold was increased from 6% to 15% (~95% specificity), the proportion of women who were considered high risk decreased (38.3% to 7.0% in the California model testing subset and 45.1% to 9.2% in the Iowa cohort) and the proportion of women who were correctly classified increased (64.3% to 89.7% in the California model testing subset and 57.1% to 88.6% in the Iowa cohort); however, the proportion of women with the disease who were considered high risk was significantly reduced (70.8% to 23.9% sensitivity for the California model testing subset and 76.7% to 24.4% sensitivity for the Iowa cohort).

The ability of the risk prediction model to correctly classify women in different racial/ethnic groups as low risk or high risk of developing GDM was also assessed (Table 3). Using the 6% threshold, 27.3% and 40.0% of White, not Hispanic women within the California model testing subset and Iowa cohort, respectively, were classified as high risk. The model was less sensitive and more specific among White, not Hispanic women in the California model testing subset than the Iowa cohort (California: sensitivity: 54.6%, specificity: 74.2%; Iowa: sensitivity: 73.9%, specificity: 61.4%) but correctly classified more women (California: 73.2%; Iowa: 61.8%).

Overall, 31.4%, 20.9%, 88.0%, 58.4%, and 37.6% of Hispanic, Black, Asian, Hawaiian/Pacific Islander women, and women in other racial groups were classified as high risk within the California model testing subset. The sensitivity and specificity of the model varied between racial/ethnic groups. Moderate values were observed among Hispanic women (sensitivity: 65.0%, specificity: 70.6%) and women in other racial groups (sensitivity: 67.3%, specificity: 64.3%). The model was less sensitive among Black women (49.3%) and more sensitive among Asian (96.3%) and Hawaiian/Pacific Islander women (80.7%). This correlates with the prevalence of GDM in these groups (lower for Black women and higher for Asian and Hawaiian/Pacific Islander women [32]). Using this risk stratification strategy, 70.3% Hispanic 79.0% Black, 23.1% Asian, 46.9% Hawaiian/Pacific Islander, and 64.5% of women in other racial groups were correctly classified.

## Discussion

### Main findings

Using a large, racial- and ethnically-diverse cohort of nulliparous women, we rigorously developed and internally and externally validated a clinical model for preconception and early pregnancy risk prediction of GDM based on clinical risk factors. The developed model, including five well-established risk factors, had moderate predictive performance among all nulliparous women, with AUCs of 0.732 and 0.710 in the internal and external cohorts, sensitivities of 70.8% and 76.7%, and correct classification of 64.3% and 57.1%, respectively. When examining the model performance in each racial/ethnic group separately, the model showed the strongest predictive ability among Hispanic and Black women in the internal validation cohort.

To our knowledge, this is the first study to develop and validate a model for preconception and early pregnancy GDM risk prediction in nulliparous women and assess model performance within specific racial/ethnic groups. The developed model includes risk factors already routinely collected by clinicians in the US, allowing for easy adaptation into existing preconception and prenatal care practice and screening programs. Importantly, our findings suggest that estimation of a woman’s risk for GDM through model-based incorporation of risk factors accurately identifies those at high risk who could benefit most from preconception or early pregnancy preventive intervention. This is especially true for Hispanic and Black women for which prediction models have not been previously validated.

### Strengths and limitations

This study is strengthened by the use of a large, racially- and ethnically-diverse, contemporary cohort for model development. The developed model is based on maternal characteristics that are routinely collected by clinicians and is available in an online, user-friendly format allowing for use in a variety of platforms to inform individuals of their risk of GDM and guide preconception and early pregnancy intervention strategies targeted towards those women at the highest risk for developing GDM. The use of a large dataset allowed for us to examine whether the model was more accurate for some subgroups than others.

Several models have been proposed for preconception or early pregnancy prediction of GDM. These models have shown moderate-to-good discriminative ability (AUCs ranging from 0.64–0.89) and have included, on average, five demographic and clinical risk factors such as maternal age, gestational age at sampling, BMI, history of gestational diabetes mellitus, family history of diabetes mellitus, race/ethnicity, prior poor obstetric outcome, history of macrosomia, diet, physical activity, and PCOS diagnosis.[25,33–43] However, previous models for early risk prediction of GDM within diverse populations have only assessed the predictive accuracy of their model across the entire cohort with no subgroup analysis,[25,33,34] which can mask model deficiencies.[44] The generalizability of other proposed models are limited due to the use of homogenous populations for model development.[35–40,43] No previous studies, to our knowledge, have assessed the predictive accuracy of their model among racial/ethnic subgroups, which may contribute to the etiology and severity of the disease.[45]

Previous studies have shown that nulliparous women are at higher risk for adverse birth outcomes than multiparous women,[17] indicating that these women may have unique risk profiles. As the relative importance of certain risk factors for GDM, such as age, smoking, infertility, hypertensive disorders of pregnancy, and socioeconomic status, differ among nulliparous and multiparous women,[17,46,47] it is important to develop separate models for these groups of women in order to more accurately predict GDM risk. To date, only one previous study has developed a model for predicting GDM risk among nulliparous women.[40] While the discriminative ability of the model was slightly higher compared to our model (AUC 0.79 vs. AUC 0.73), the model was developed using a smaller, racially/ethnically homogenous population of Australian women and included variables not routinely collected by most clinicians (i.e., dietary intake (based on a food frequency questionnaire) and physical activity (based on hours per week spent on moderate-vigorous activity)).

Race is a strong independent risk factor for GDM, with higher rates observed among Asians, Pacific Islanders, North African, and Hispanic women than Caucasian women.[45,48] Significant variation in the association between race/ethnicity and GDM risk by BMI categories has been observed, with Asian and Filipina women having increased risk of GDM at lower BMIs compared with Caucasian and African American women.[45] While the biological mechanisms behind this observation are largely unknown, it has been suggested that higher percentages of body fat and more visceral adipose tissue for a given BMI among Asian women in comparison with other racial/ethnic groups and/or differences in genetic predisposition may be contributing to this finding.[45,49–51] Incorporation of body fat and visceral adipose tissue measurements may increase the predictive accuracy of our model within Asian women and should be assessed in future studies.

Distinguishing between GDM and pre-existing diabetes is challenging as many at-risk women do not undergo screening for diabetes mellitus prior to conception.[52] However, it has been estimated that of the 6–9% of pregnancies affected by diabetes, about 90% have GDM.[52–54] Another challenge to studying GDM is that there is not a single, agreed-upon method to diagnose this condition. Different diagnostic criteria could have been used between the California and Iowa cohorts, leading to differences in GDM prevalence. As the two-step approach to diagnostic testing for GDM recommended by the American College of Obstetricians and Gynecologists is most commonly used in the United States,[8,52] it is reasonable to assume that most physicians adhered to these guidelines. Additionally, some women with GDM may have been missed; however, the number of missed diagnoses is likely to be small since universal screening at 24–28 weeks gestation is standard of care.[8,9] While we were unable to confirm GDM diagnoses within the California sample, the combined use of hospital discharge and birth certificate data has been shown to be an accurate source for GDM ascertainment.[55]

Another limitation was the exclusion of women who did not have complete data for the covariates of interest. Although the use of complete case analysis may lend itself to bias, the bias produced is likely to be minor as only a small percentage of women in both the California and Iowa cohorts had missing predictor data. Our final model included age at delivery, which may not be accurately captured for women using our proposed risk calculator prior to pregnancy. However, it is likely that women receiving risk estimates will be able to closely estimate their age at delivery, inducing a negligible effect on risk estimation. We were unable to validate models among American Indian/Alaska Native women in the California model testing subset or any racial/group other than White, not Hispanic in the Iowa cohort due to small sample sizes. Further external validation of the developed model within these racial/ethnic groups is needed.

We were able to both internally and externally validate our model to assess its predictive performance and generalizability across health care settings and populations. The next step is to evaluate whether implementation of the risk prediction model in clinical practice improves maternal and infant outcomes through personalized prevention or treatment strategies. This would typically involve an impact study, ideally a randomized controlled trial.[44]

### Interpretation

At present, there is no international consensus on the optimal screening strategy for GDM. Early pregnancy screening for GDM or undiagnosed type 2 diabetes (i.e., <24 weeks gestation) based on clinical risk factors is recommended by some expert groups with the goal of identifying those who should undergo early glucose testing and diagnosis.[52,56] However, clinical tools aimed at identifying women prior to or early in pregnancy who are at high risk for GDM and may benefit most from preventive intervention are not currently used in practice. Although the prevalence of GDM has been reported to be high (~16%) among women who are giving birth for the first time,[57] these women are particularly vulnerable to non-compliance with risk-based GDM screening guidelines.[16] Early pregnancy assessment of risk factors for GDM in nulliparous women may facilitate early streamlined antenatal care, preventive intervention, improved patient experience, and enhanced short-and long-term clinical outcomes for mother and baby.[58]

Pregnancy is a critical period during a woman’s life when adoption of a healthy lifestyle should become a priority.[3,59] As physical activity and maintaining a well-balanced diet have minimal risks, both are recommended for women with uncomplicated pregnancies.[59,60] Implementation of lifestyle interventions prior to or earlier in pregnancy (before the 20th week of gestation) is of increased importance for women at high risk for GDM, as these interventions could limit gestational weight gain and reduce the risk of developing GDM later in pregnancy.[10–12] While most women report having knowledge of appropriate diet and exercise regimens for pregnancy,[3,61] many do not meet the recommended exercise guidelines and express a desire for information on how to improve their health behaviors during pregnancy.[3,62] Mobile health technology is commonly used in the management of chronic conditions. As nearly 60% of women report the usage of a pregnancy app,[63] mobile technology could be utilized as a supplementary tool for antenatal lifestyle interventions in pregnancy.[3]

We decided *a priori* to define our risk stratification threshold based on maximization of model sensitivity and specificity, as is desirable for screening tests.[30] At the chosen predicted risk threshold of 6%, about 40% of women were classified as high risk. Most of these women could be targeted for more general intervention strategies, such as exercise and dietary support groups.[64] However, higher thresholds could be utilized to identify women at very high risk who may benefit from more comprehensive and individually-tailored lifestyle interventions delivered by dietitians, health coaches, nurses, or physicians.[11]

Because our model includes characteristics that could be estimated prior to pregnancy, it could be used by primary care providers as well as obstetricians and gynecologists who care for women prior to pregnancy. Preconception care is an opportunity to identify conditions that may be detrimental to the mother and her future children and recommend preventive behavioral, medical, or educational interventions that could improve pregnancy outcomes. Identifying women of childbearing age who are at increased risk of developing GDM aids in the promotion of optimal glucose control and health status before becoming pregnant, reducing the risk of both short and long-term maternal and infant complications.[65] Future work should focus on evaluating the clinical impact of model implementation on maternal and infant outcomes as well as the financial costs and benefits to the health care system.

## Supporting information

S1 File TRIPOD checklist. (PDF)

S1 Table Assessment of missing demographic and clinical information for women in the California model development subset (n = 771,140). (PDF)

S2 Table Demographic and clinical characteristics of nulliparous women with gestational diabetes mellitus compared to nulliparous women without gestational diabetes mellitus within the California model development subset (n = 706,659). (PDF)

S3 Table Model estimates for gestational diabetes mellitus risk prediction based on data for the entire nulliparous cohort within the California model development subset (n = 706,674). (PDF)

S4 Table Demographic and clinical characteristics of nulliparous women with gestational diabetes mellitus compared to nulliparous women without gestational diabetes mellitus within the California model testing subset (n = 352,992) and Iowa cohort (n = 3,744). (PDF)

S5 Table Assessment of missing demographic and clinical information for women in the California model testing subset (n = 385,568) and Iowa cohort (n = 4,225). (PDF)

S6 Table Characteristics of nulliparous women with glucose screening test values compared to those without test values within the Iowa cohort. (PDF)

S7 Table Demographic and clinical characteristics of White, not Hispanic nulliparous women with gestational diabetes mellitus compared to White, not Hispanic nulliparous women without gestational diabetes mellitus within the California model testing subset (n = 106,806) and Iowa cohort (n = 2,749). (PDF)

S8 Table Demographic and clinical characteristics of Hispanic nulliparous women with gestational diabetes mellitus compared to Hispanic nulliparous women without gestational diabetes mellitus within the California model testing subset (n = 148,703) and Iowa cohort (n = 232). (PDF)

S9 Table Demographic and clinical characteristics of Black nulliparous women with gestational diabetes mellitus compared to Black nulliparous women without gestational diabetes mellitus within the California model testing subset (n = 18,853) and Iowa cohort (n = 305). (PDF)

S10 Table Demographic and clinical characteristics of Asian nulliparous women with gestational diabetes mellitus compared to Asian nulliparous women without gestational diabetes mellitus within the California model testing subset (n = 51,242) and Iowa cohort (n = 323). (PDF)

S11 Table Demographic and clinical characteristics of American Indian/Alaska Native nulliparous women with gestational diabetes mellitus compared to American Indian/Alaska Native nulliparous women without gestational diabetes mellitus within the California model testing subset (n = 1,453) and Iowa cohort. (PDF)

S12 Table Demographic and clinical characteristics of Hawaiian/Pacific Islander nulliparous women with gestational diabetes mellitus compared to Hawaiian/Pacific Islander nulliparous women without gestational diabetes mellitus within the California model testing subset (n = 1,328) and Iowa cohort. (PDF)

S13 Table Demographic and clinical characteristics of nulliparous women in other racial groups with gestational diabetes mellitus compared to nulliparous women of other racial groups without gestational diabetes mellitus within the California model testing subset (n = 24,607) and Iowa cohort. (PDF)

S14 Table Accuracy of initial and final models among all nulliparous women in the California model testing subset and Iowa cohort. (PDF)

S15 Table Clinical scenarios demonstrating the use of the final risk prediction model for calculating predicted risk of gestational diabetes mellitus. (PDF)

S16 Table Performance of different risk stratification strategies within the California model testing subset and Iowa cohort. (PDF)
