# Supplementary material for: Development and validation of a clinical model for preconception and early pregnancy risk prediction of gestational diabetes mellitus in nulliparous women
Source: PLoS One. 2019 Apr 12;14(4):e0215173. doi: 10.1371/journal.pone.0215173 (PMC6461273; doi:10.1371/journal.pone.0215173)
Supplement: S1 Table — (PDF) [file pone.0215173.s002.pdf]

**S1 Table. Assessment of missing demographic and clinical information for women in the California model development subset (n=771,140).**

| | Subjects with Missing Information | Subjects with Complete Data (n=706,674) | Subjects with at Least One Missing Data Value (n=64,466) |
|---|---|---|---|
| **Race/ethnicity** | 0 | | |
| White, not Hispanic | | 213,512 (30.2) | 14,707 (22.8) |
| Hispanic | | 297,894 (42.2) | 30,623 (47.5) |
| Black | | 37,479 (5.3) | 4,562 (7.1) |
| Asian | | 101,782 (14.4) | 9,916 (15.4) |
| AI/AN | | 2,981 (0.4) | 314 (0.5) |
| H/PI | | 2,645 (0.4) | 314 (0.5) |
| Other racial group[†] | | 50,381 (7.1) | 4,030 (6.3) |
| **Age at delivery (years)**[‡] | 50 | 25.9 (6.3) | 25.3 (6.2) |
| **Pre-pregnancy BMI (kg/m$^2$)** [‡] | 64,431 | 24.6 (5.1) | 24.2 (4.4) |
| **Family history of diabetes** | 0 | 6,229 (0.9) | 394 (0.6) |
| **Pre-existing hypertension** | 0 | 7,778 (1.1) | 1,212 (1.9) |

[†]Includes two or more races and race unknown.

[‡]Data are expressed as mean (SD). All other variables are expressed as n (%).
