# Supplementary material for: Development and validation of a clinical model for preconception and early pregnancy risk prediction of gestational diabetes mellitus in nulliparous women
Source: PLoS One. 2019 Apr 12;14(4):e0215173. doi: 10.1371/journal.pone.0215173 (PMC6461273; doi:10.1371/journal.pone.0215173)
Supplement: S2 Table — (PDF) [file pone.0215173.s003.pdf]

S2 Table. Demographic and clinical characteristics of nulliparous women with gestational diabetes mellitus compared to nulliparous women without gestational diabetes mellitus within the California model development subset (n=706,659).

	No GDM n (%)	GDM n (%)	OR (95% CI)	aOR (95% CI)
**Sample Size**	**662,411 (93.7)**	**44,248 (6.3)**		
**Race/ethnicity**				
White, not Hispanic	202,538 (30.6)	10,972 (24.8)	REF	REF
Hispanic	281,677 (42.5)	16,216 (36.7)	1.06 (1.04, 1.09)*	1.45 (1.41, 1.49)*
Black	35,901 (5.4)	1,577 (3.6)	0.81 (0.77, 0.86)*	0.96 (0.91, 1.02)
Asian	89,715 (13.5)	12,065 (27.3)	2.48 (2.42, 2.55)*	2.93 (2.85, 3.02)*
AI/AN	2,835 (0.4)	142 (0.3)	0.93 (0.78, 1.10)	1.15 (0.96, 1.36)
H/PI	2,401 (0.4)	244 (0.6)	1.88 (1.64, 2.14)*	1.90 (1.65, 2.18)*
Other racial group[†]	47,344 (7.2)	3,032 (6.9)	1.18 (1.13, 1.23)*	1.38 (1.32, 1.44)*
**Age at delivery (years)**[‡a]	25.7 (6.3)	29.4 (6.2)	1.09 (1.09, 1.09)*	1.09 (1.09, 1.09)*
**Expected payer for delivery**				
Government	298,994 (45.1)	15,474 (35.0)	0.64 (0.63, 0.65)*	1.07 (1.04, 1.09)*
Private	340,500 (51.4)	27,551 (62.3)	REF	REF
Other	22,917 (3.5)	1,223 (2.8)	0.66 (0.62, 0.70)*	0.75 (0.71, 0.80)*
**Smoked during pregnancy**	22,651 (3.4)	1,280 (2.9)	0.84 (0.80, 0.89)*	1.08 (1.02, 1.15)
**Pre-pregnancy BMI (kg/m^2)**[‡b]	24.5 (5.0)	26.9 (6.1)	1.08 (1.08, 1.08)*	1.10 (1.09, 1.10)*
**Family history of diabetes**	5,465 (0.8)	764 (1.7)	2.11 (1.96, 2.28)*	1.93 (1.78, 2.09)*
**PCOS diagnosis**	1,233 (0.2)	341 (0.8)	4.17 (3.69, 4.70)*	2.11 (1.86, 2.41)*
**Pre-existing hypertension**	6,346 (1.0)	1,432 (3.2)	3.46 (3.26, 3.67)*	1.63 (1.53, 1.73)*
**Pre-existing dyslipidemia**	1,202 (0.2)	303 (0.7)	3.79 (3.34, 4.30)*	1.74 (1.52, 2.00)*
**Personal history of CVD**	1,129 (0.2)	110 (0.3)	1.46 (1.20, 1.78)*	1.05 (0.86, 1.29)
**Assisted reproductive technology use**	5,016 (0.8)	828 (1.9)	2.50 (2.32, 2.70)*	1.17 (1.08, 1.26)*
**Personal history of miscarriage**	1,904 (0.3)	202 (0.5)	1.59 (1.38, 1.84)*	1.26 (1.08, 1.46)

GDM, gestational diabetes mellitus; OR, odds ratio; aOR, adjusted odds ratio; CI, confidence interval; REF, reference group; AI/AN, American Indian/Alaska Native; H/PI, Hawaiian/Pacific Islander; BMI, body mass index; PCOS, polycystic ovarian syndrome; CVD, cardiovascular disease. Odds ratios and two- sided *P* values were estimated using univariate logistic regression. Adjusted odds ratios and two-sided *P* values were estimated using multivariate logistic regression. Each variable was adjusted for all other variables within the table.

[†]Includes two or more races and race unknown.

[‡]Data are expressed as mean (SD).

[a]Odds ratios were calculated per year of age.

[b]Odds ratios were calculated per kg/m^2.

*Two-sided *P* <0.001.
