# Supplementary material for: Development and validation of a clinical model for preconception and early pregnancy risk prediction of gestational diabetes mellitus in nulliparous women
Source: PLoS One. 2019 Apr 12;14(4):e0215173. doi: 10.1371/journal.pone.0215173 (PMC6461273; doi:10.1371/journal.pone.0215173)
Supplement: S3 Table — (PDF) [file pone.0215173.s004.pdf]

S3 Table. Model estimates for gestational diabetes mellitus risk prediction based on data for the entire nulliparous cohort within the California model development subset (n=706,674).

Variable	Final Model		
	**β (SE)**	**Wald χ^2**	***P* value**
Race/ethnicity		5,866.6 (global)	<0.001* (global)
White, not Hispanic	REF	REF	REF
Hispanic	0.376 (0.014)	762.1	<0.001*
Black	-0.034 (0.029)	1.4	0.241
Asian	1.066 (0.014)	5,468.5	<0.001*
AI/AN	0.144 (0.089)	2.6	0.105
H/PI	0.639 (0.071)	82.0	<0.001*
Other racial group†	0.328 (0.022)	228.4	<0.001*
Age at delivery (years)	0.087 (0.001)	10,589.0	<0.001*
Pre-pregnancy BMI (kg/m^2)	0.093 (0.001)	11,024.2	<0.001*
Family history of diabetes	0.688 (0.041)	283.2	<0.001*
Pre-existing hypertension	0.508 (0.032)	255.2	<0.001*

SE, standard error; REF, reference; AI/AN, American Indian/Alaska Native; H/PI, Hawaiian/Pacific Islander; BMI, body mass index; PCOS, polycystic ovarian syndrome; CVD, cardiovascular disease.
*Two-sided *P* <0.001
†Includes two or more races and race unknown.
