# Supplementary material for: Development and validation of a clinical model for preconception and early pregnancy risk prediction of gestational diabetes mellitus in nulliparous women
Source: PLoS One. 2019 Apr 12;14(4):e0215173. doi: 10.1371/journal.pone.0215173 (PMC6461273; doi:10.1371/journal.pone.0215173)
Supplement: S4 Table — (PDF) [file pone.0215173.s005.pdf]

S4 Table. Demographic and clinical characteristics of nulliparous women with gestational diabetes mellitus compared to nulliparous women without gestational diabetes mellitus within the California model testing subset (n=352,992) and Iowa cohort (n=3,744).

	**California Model Testing Subset**				**Iowa Cohort**			
	**No GDM n (%)**	**GDM n (%)**	**OR (95% CI)**	**aOR (95% CI)**	**No GDM n (%)**	**GDM n (%)**	**OR (95% CI)**	**aOR (95% CI)**
**Sample Size**	**330,798 (93.7)**	**22,194 (6.3)**			**3,585 (95.8)**	**159 (4.3)**		
**Race/ethnicity**								
White, not Hispanic	101,257 (30.6)	5,549 (25.0)	REF	REF	2,647 (73.8)	102 (64.2)	REF	REF
Hispanic	140,618 (42.5)	8,085 (36.4)	1.05 (1.01, 1.09)	1.44 (1.38, 1.50)*	218 (6.1)	14 (8.8)	1.67 (0.94, 2.96)	2.30 (1.24, 4.27)
Black	18,133 (5.5)	720 (3.2)	0.73 (0.67, 0.78)*	0.86 (0.79, 0.94)*	293 (8.2)	12 (7.6)	1.06 (0.58, 1.96)	1.43 (0.74, 2.76)
Asian	45,087 (13.6)	6,155 (27.7)	2.49 (2.40, 2.59)*	2.97 (2.85, 3.09)*	298 (8.3)	25 (15.7)	2.18 (1.38, 3.43)*	2.84 (1.75, 4.60)*
AI/AN	1,359 (0.4)	94 (0.4)	1.26 (1.02, 1.56)	1.62 (1.30, 2.02)*	--	--	--	--
H/PI	1,214 (0.4)	114 (0.5)	1.71 (1.41, 2.08)*	1.77 (1.45, 2.16)*	--	--	--	--
Other racial group[†]	23,130 (7.0)	1,477 (6.7)	1.17 (1.10, 1.24)*	1.36 (1.28, 1.44)*	119 (3.3)	--	--	--
**Age at delivery (years)**[‡a]	25.7 (6.3)	29.4 (6.1)	1.09 (1.09, 1.10)*	1.09 (1.09, 1.10)*	27.6 (5.3)	29.8 (5.4)	1.08 (1.05, 1.11)*	1.08 (1.05, 1.11)*
**Expected payer for delivery**								
Government	149,014 (45.1)	7,597 (34.2)	0.62 (0.60, 0.64)*	1.04 (1.01, 1.08)	845 (23.6)	--	0.82 (0.55, 1.23)	0.88 (0.54, 1.41)
Private	170,191 (51.5)	14,026 (63.2)	REF	REF	2,656 (74.1)	122 (76.7)	REF	REF
Other	11,593 (3.5)	571 (2.6)	0.60 (0.55, 0.65)*	0.68 (0.62, 0.74)*	84 (2.3)	--	--	--
**Smoked during pregnancy**	11,284 (3.4)	620 (2.8)	0.81 (0.75, 0.88)*	1.07 (0.98, 1.17)	381 (10.6)	17 (10.7)	1.01 (0.60, 1.68)	1.25 (0.72, 2.18)
**Pre-pregnancy BMI (kg/m^2)** [‡b]	24.5 (5.0)	26.9 (6.1)	1.08 (1.08, 1.08)*	1.10 (1.09, 1.10)*	27.3 (6.6)	30.6 (8.1)	1.06 (1.04, 1.08)*	1.07 (1.05, 1.09)*
**Family history of diabetes**	2,717 (0.8)	390 (1.8)	2.16 (1.94, 2.41)*	1.92 (1.71, 2.15)*	32 (0.9)	--	--	--
**PCOS diagnosis**	565 (0.2)	186 (0.8)	4.95 (4.19, 5.85)*	2.36 (1.98, 2.83)*	161 (4.5)	10 (6.3)	1.43 (0.74, 2.76)	0.86 (0.43, 1.72)
**Pre-existing hypertension**	3,090 (0.9)	733 (3.3)	3.62 (3.34, 3.93)*	1.68 (1.54, 1.84)*	--	--	--	--
**Pre-existing dyslipidemia**	582 (0.2)	155 (0.7)	3.99 (3.34, 4.77)*	1.83 (1.51, 2.22)*	58 (1.6)	--	--	
**Personal history of CVD**	542 (0.2)	47 (0.2)	1.29 (0.96, 1.74)	0.91 (0.66, 1.24)	25 (0.7)	--	--	--
**Assisted reproductive technology use**	2,484 (0.8)	401 (1.8)	2.43 (2.19, 2.71)*	1.12 (1.00, 1.25)	--	--	--	--
**Personal history of miscarriage**	945 (0.3)	99 (0.5)	1.56 (1.27, 1.93)*	1.24 (1.00, 1.54)	15 (0.4)	--	--	--

GDM, gestational diabetes mellitus; OR, odds ratio; aOR, adjusted odds ratio; CI, confidence interval; REF, reference group; AI/AN, American Indian/Alaska Native; H/PI, Hawaiian/Pacific Islander; BMI, body mass index; PCOS, polycystic ovarian syndrome; CVD, cardiovascular disease.

Odds ratios and two-sided *P* values were estimated using univariate logistic regression. Adjusted odds ratios and two-sided *P* values were estimated using multivariate logistic regression. Each variable was adjusted for all other variables within the table.

[†] Includes two or more races and race unknown.

[‡] Data are expressed as mean (SD).

[a] Odds ratios were calculated per year.

[b] Odds ratios were calculated per kg/m^2.

*Two-sided P <0.001.
