# Supplementary material for: Development and validation of a clinical model for preconception and early pregnancy risk prediction of gestational diabetes mellitus in nulliparous women
Source: PLoS One. 2019 Apr 12;14(4):e0215173. doi: 10.1371/journal.pone.0215173 (PMC6461273; doi:10.1371/journal.pone.0215173)
Supplement: S5 Table — (PDF) [file pone.0215173.s006.pdf]

S5 Table. **Assessment of missing demographic and clinical information for women in the California model testing subset (n=385,568) and Iowa cohort (n=4,225).**

	California Model Testing Subset			Iowa Cohort		
	**Subjects with Missing Information**	**Subjects with Complete Data (n=353,003)**	**Subjects with at Least One Missing Data Value (n=32,565)**	**Subjects with Missing Information**	**Subjects with Complete Data (n=4,145)**	**Subjects with at Least One Missing Data Value (n=80)**
**Race/ethnicity**	0			0		
White, not Hispanic		106,808 (30.3)	7,494 (23.0)		3,063 (73.9)	51 (63.8)
Hispanic		148,703 (42.1)	15,300 (47.0)		246 (5.9)	--
Black		18,853 (5.3)	2,311 (7.1)		312 (7.5)	13 (16.3)
Asian		51,246 (14.5)	5,049 (15.5)		372 (9.0)	--
AI/AN		1,455 (0.4)	172 (0.5)		--	--
H/PI		1,328 (0.4)	169 (0.5)		--	--
Other racial group†		24,610 (7.0)	2,070 (6.4)		135 (3.3)	--
**Age at delivery (years)**‡	9	25.9 (6.3)	25.4 (6.3)	0	27.8 (5.3)	26.2 ± 5.7
**Pre-pregnancy BMI (kg/m^2)** ‡	32,559	24.6 (5.1)	21.6 (2.7)	80	27.3 (6.6)	*
**Family history of diabetes**	0	3,107 (0.9)	184 (0.6)	0	38 (0.9)	--
**Pre-existing hypertension**	0	3,823 (1.1)	571 (1.8)	0	--	--

†Includes two or more races and race unknown.

‡Data are expressed as mean (SD). All other variables are expressed as n (%).

-- Data suppressed (n <10).

*Mean (SD) could not be calculated (all subjects were missing BMI data).
