# Supplementary material for: Development and validation of a clinical model for preconception and early pregnancy risk prediction of gestational diabetes mellitus in nulliparous women
Source: PLoS One. 2019 Apr 12;14(4):e0215173. doi: 10.1371/journal.pone.0215173 (PMC6461273; doi:10.1371/journal.pone.0215173)
Supplement: S6 Table — (PDF) [file pone.0215173.s007.pdf]

S6 Table. Characteristics of nulliparous women with glucose screening test values compared to those without test values within the Iowa cohort.

	Subjects with Glucose Screening Test Values (n= 4,225)	Subjects with Missing Glucose Screening Test Values (n= 1,147)	*P* value
**GDM**	181 (4.3)	NA	
**Race/ethnicity**			0.001
White, not Hispanic	3,114 (73.7)	901 (78.6)	
Hispanic	248 (5.9)	86 (7.5)	
Black	325 (7.7)	78 (6.8)	
Asian	381 (9.0)	31 (2.7)	
AI/AN	--	--	
H/PI	--	--	
Other racial group†	140 (3.3)	39 (3.4)	
**Age at delivery (years)**‡	27.8 (5.3)	25.7 (5.8)	<0.001
**Expected payer for delivery**			<0.001
Government	909 (23.8)	456 (43.3)	
Private	2,822 (73.9)	558 (53.0)	
Other	90 (2.4)	39 (3.7)	
Missing	404	94	
**Smoked during pregnancy**	441 (10.4)	242 (21.1)	<0.001
**Pre-pregnancy BMI (kg/m^2)**‡	27.3 (6.6)	31.0 (7.5)	<0.001
Missing	80	77	
**Family history of diabetes**	38 (0.9)	--	0.215
**PCOS diagnosis**	199 (4.7)	28 (2.4)	<0.001
**Pre-existing hypertension**	--	--	0.959
**Pre-existing dyslipidemia**	76 (1.8)	--	0.001
**Personal history of CVD**	28 (0.7)	--	0.598
**Assisted reproductive technology use**	--	--	0.953
**Personal history of miscarriage**	16 (0.4)	--	0.154

GDM, gestational diabetes mellitus; AI/AN, American Indian/Alaska Native; H/PI, Hawaiian/Pacific Islander; SD, standard deviation; BMI, body mass index; PCOS, polycystic ovarian syndrome; CVD, cardiovascular disease.
NA, not applicable (GDM status could not be determined as all subjects were missing glucose screening test values).
†Includes two or more races and race unknown.
‡Data are expressed as mean (SD). All other variables are expressed as n (%).
Two-sided *P* values were calculated using univariate logistic regression.
-- Data suppressed (n <10).
