# Supplementary material for: Development and validation of a clinical model for preconception and early pregnancy risk prediction of gestational diabetes mellitus in nulliparous women
Source: PLoS One. 2019 Apr 12;14(4):e0215173. doi: 10.1371/journal.pone.0215173 (PMC6461273; doi:10.1371/journal.pone.0215173)
Supplement: S7 Table — (PDF) [file pone.0215173.s008.pdf]

S7 Table. Demographic and clinical characteristics of White, not Hispanic nulliparous women with gestational diabetes mellitus compared to White, not Hispanic nulliparous women without gestational diabetes mellitus within the California model testing subset (n=106,806) and Iowa cohort (n=2,749).

	California Model Testing Subset				Iowa Cohort			
	No GDM n (%)	GDM n (%)	OR (95% CI)	aOR (95% CI)	No GDM n (%)	GDM n (%)	OR (95% CI)	aOR (95% CI)
**Sample Size**	**101,257 (94.8)**	**5,549 (5.2)**			**2,647 (96.3)**	**102 (3.7)**		
**Age at delivery (years)**[ǂa]	28.1 (6.0)	30.6 (6.0)	1.07 (1.07, 1.07)*	1.08 (1.07, 1.08)*	28.1 (5.0)	30.2 (5.3)	1.08 (1.04, 1.12)*	1.09 (1.05, 1.13)*
**Expected payer for delivery**								
Government	24,528 (24.2)	1,130 (20.4)	0.80 (0.75, 0.85)*	1.13 (1.06, 1.22)	483 (18.3)	20 (19.6)	1.07 (0.65, 1.76)	1.04 (0.59, 1.85)
Private	74,310 (73.4)	4,300 (77.5)	REF	REF	2,122 (80.2)	82 (80.4)	REF	REF
Other	2,419 (2.4)	119 (2.1)	0.85 (0.71, 1.03)	0.94 (0.78, 1.14)	42 (1.6)	--	--	--
**Smoked during pregnancy**	6,000 (5.9)	321 (5.8)	0.98 (0.87, 1.10)	1.14 (1.01, 1.29)	308 (11.6)	16 (15.7)	1.41 (0.82, 2.44)	1.54 (0.84, 2.80)
**Pre-pregnancy BMI (kg/m^2)**[ǂb]	24.2 (4.9)	27.4 (6.4)	1.10 (1.10, 1.11)*	1.10 (1.10, 1.11)*	27.5 (6.6)	32.3 (8.4)	1.08 (1.06, 1.10)*	1.08 (1.05, 1.10)*
**Family history of diabetes**	560 (0.6)	69 (1.2)	2.27 (1.76, 2.91)*	1.85 (1.42, 2.40)*	23 (0.9)	--	--	--
**PCOS diagnosis**	237 (0.2)	52 (0.9)	4.03 (2.98, 5.45)*	2.05 (1.49, 2.82)*	131 (5.0)	--	1.42 (0.64, 3.11)	0.78 (0.34, 1.79)
**Pre-existing hypertension**	1,157 (1.1)	207 (3.7)	3.35 (2.88, 3.90)*	1.58 (1.35, 1.85)*	--	--	--	--
**Pre-existing dyslipidemia**	207 (0.2)	41 (0.7)	3.63 (2.60, 5.09)*	1.80 (1.26, 2.58)	52 (2.0)	--	--	--
**Personal history of CVD**	284 (0.3)	19 (0.3)	1.22 (0.77, 1.95)	0.84 (0.52, 1.36)	24 (0.9)	--	--	--
**Assisted reproductive technology use**	1,414 (1.4)	157 (2.8)	2.06 (1.74, 2.43)*	1.22 (1.02, 1.45)	--	--	--	--
**Personal history of miscarriage**	247 (0.2)	21 (0.4)	1.55 (0.99, 2.43)	1.32 (0.83, 2.09)	10 (0.4)	--	--	--

GDM, gestational diabetes mellitus; OR, odds ratio; aOR, adjusted odds ratio; CI, confidence interval; REF, reference group; BMI, body mass index; PCOS, polycystic ovarian syndrome; CVD, cardiovascular disease

Odds ratios and two-sided *P* values were estimated using univariate logistic regression. Adjusted odds ratios and two-sided *P* values were estimated using multivariate logistic regression. Each variable was adjusted for all other variables within the table.

[ǂ]Data are expressed as mean (SD).

[a]Odds ratios were calculated per year.

[b]Odds ratios were calculated per kg/m^2.

*Two-sided *P* <0.001.

-- Data suppressed (n <10); OR and aOR not calculated.
