# Supplementary material for: Development and validation of a clinical model for preconception and early pregnancy risk prediction of gestational diabetes mellitus in nulliparous women
Source: PLoS One. 2019 Apr 12;14(4):e0215173. doi: 10.1371/journal.pone.0215173 (PMC6461273; doi:10.1371/journal.pone.0215173)
Supplement: S8 Table — (PDF) [file pone.0215173.s009.pdf]

S8 Table. Demographic and clinical characteristics of Hispanic nulliparous women with gestational diabetes mellitus compared to Hispanic nulliparous women without gestational diabetes mellitus within the California model testing subset (n=148,703) and Iowa cohort (n=232).

	California Model Testing Subset				Iowa Cohort			
	**No GDM n (%)**	**GDM n (%)**	**OR (95% CI)**	**aOR (95% CI)**	**No GDM n (%)**	**GDM n (%)**	**OR (95% CI)**	**aOR (95% CI)**
**Sample Size**	**140,618 (94.6)**	**8,085 (5.4)**			**218 (94.0)**	**14 (6.0)**		
**Age at delivery (years)**[ǂa]	23.1 (5.5)	27.2 (6.2)	1.12 (1.12, 1.12)*	1.11 (1.11, 1.12)*	23.7 (5.6)	28.1 (6.2)	1.13 (1.03, 1.23)	1.20 (1.06, 1.35)
**Expected payer for delivery**								
Government	92,262 (65.6)	4,579 (56.6)	0.65 (0.62, 0.68)*	1.12 (1.06, 1.17)*	114 (52.3)	--	--	--
Private	43,067 (30.6)	3,288 (40.7)	REF	REF	83 (38.1)	--	REF	REF
Other	5,289 (3.8)	218 (2.7)	0.54 (0.47, 0.62)*	0.70 (0.61, 0.81)*	21 (9.6)	--	--	--
**Smoked during pregnancy**	2,631 (1.9)	140 (1.7)	0.92 (0.78, 1.10)	0.92 (0.77, 1.10)	--	--	--	--
**Pre-pregnancy BMI (kg/m^2)**[ǂb]	25.2 (5.1)	28.4 (6.0)	1.11 (1.10, 1.11)*	1.09 (1.09, 1.10)*	28.2 (6.6)	27.7 (4.8)	0.99 (0.91, 1.08)	0.96 (0.86, 1.07)
**Family history of diabetes**	1,614 (1.2)	180 (2.2)	1.96 (1.68, 2.29)*	1.70 (1.44, 2.00)*	--	--	--	--
**PCOS diagnosis**	175 (0.1)	62 (0.8)	6.21 (4.65, 8.31)*	2.38 (1.74, 3.25)*	--	--	--	--
**Pre-existing hypertension**	907 (0.7)	231 (2.9)	4.53 (3.91, 5.24)*	1.69 (1.45, 1.99)*	--	--	--	--
**Pre-existing dyslipidemia**	208 (0.2)	56 (0.7)	4.72 (3.51, 6.34)*	1.82 (1.32, 2.51)*	--	--	--	--
**Personal history of CVD**	126 (0.1)	11 (0.1)	1.52 (0.82, 2.82)	0.85 (0.45, 1.62)	--	--	--	--
**Assisted reproductive technology use**	340 (0.2)	78 (1.0)	4.02 (3.14, 5.15)*	1.23 (0.94, 1.60)	--	--	--	--
**Personal history of miscarriage**	454 (0.3)	41 (0.5)	1.57 (1.14, 2.17)	1.19 (0.85, 1.65)	--	--	--	--

GDM, gestational diabetes mellitus; OR, odds ratio; aOR, adjusted odds ratio; CI, confidence interval; REF, reference group; BMI, body mass index; PCOS, polycystic ovarian syndrome; CVD, cardiovascular disease

Odds ratios and two-sided *P* values were estimated using univariate logistic regression. Adjusted odds ratios and two-sided *P* values were estimated using multivariate logistic regression. Each variable was adjusted for all other variables within the table.

[ǂ]Data are expressed as mean (SD).

[a]Odds ratios were calculated per year.

[b]Odds ratios were calculated per kg/m^2.

*Two-sided *P* <0.001.

-- Data suppressed (n <10); OR and aOR not calculated.
