# Supplementary material for: Development and validation of a clinical model for preconception and early pregnancy risk prediction of gestational diabetes mellitus in nulliparous women
Source: PLoS One. 2019 Apr 12;14(4):e0215173. doi: 10.1371/journal.pone.0215173 (PMC6461273; doi:10.1371/journal.pone.0215173)
Supplement: S9 Table — (PDF) [file pone.0215173.s010.pdf]

S9 Table. Demographic and clinical characteristics of Black nulliparous women with gestational diabetes mellitus compared to Black nulliparous women without gestational diabetes mellitus within the California model testing subset (n=18,853) and Iowa cohort (n=305).

	California Model Testing Subset				Iowa Cohort			
	**No GDM n (%)**	**GDM n (%)**	**OR (95% CI)**	**aOR (95% CI)**	**No GDM n (%)**	**GDM n (%)**	**OR (95% CI)**	**aOR (95% CI)**
**Sample Size**	**18,133 (96.2)**	**720 (3.8)**			**293 (96.1)**	**12 (3.9)**		
**Age at delivery (years)**[ǂa]	23.4 (5.7)	27.1 (6.6)	1.10 (1.08, 1.11)*	1.08 (1.07, 1.10)*	23.8 (5.2)	26.7 (6.7)	1.09 (0.99, 1.19)	1.09 (0.99, 1.20)
**Expected payer for delivery**								
Government	10,693 (59.0)	352 (48.9)	0.64 (0.53, 0.74)*	1.01 (0.85, 1.19)	201 (68.6)	--	--	--
Private	6,788 (37.4)	351 (48.8)	REF	REF	83 (28.3)	--	REF	REF
Other	652 (3.6)	17 (2.4)	0.50 (0.31, 0.83)	0.64 (0.39, 1.06)	--	--	--	--
**Smoked during pregnancy**	984 (5.4)	37 (5.1)	0.94 (0.67, 1.32)	0.93 (0.65, 1.31)	39 (13.3)	--	--	--
**Pre-pregnancy BMI (kg/m^2)**[ǂb]	25.7 (5.8)	29.1 (6.5)	1.09 (1.08, 1.10)*	1.07 (1.06, 1.09)*	28.4 (7.3)	32.1 (9.0)	1.06 (0.99, 1.12)	1.07 (1.00, 1.15)
**Family history of diabetes**	106 (0.6)	15 (2.1)	3.62 (2.10, 6.25)*	2.85 (1.60, 5.08)*	--	--	--	--
**PCOS diagnosis**	23 (0.1)	--	--	--	--	--	--	--
**Pre-existing hypertension**	333 (1.8)	48 (6.7)	3.82 (2.79, 5.22)*	1.74 (1.25, 2.44)*	--	--	--	--
**Pre-existing dyslipidemia**	39 (0.2)	--	--	--	--	--	--	--
**Personal history of CVD**	44 (0.2)	--	--	--	--	--	--	--
**Assisted reproductive technology use**	58 (0.3)	--	--	--	--	--	--	--
**Personal history of miscarriage**	59 (0.3)	--	--	--	--	--	--	--

GDM, gestational diabetes mellitus; OR, odds ratio; aOR, adjusted odds ratio; CI, confidence interval; REF, reference group; BMI, body mass index; PCOS, polycystic ovarian syndrome; CVD, cardiovascular disease

Odds ratios and two-sided *P* values were estimated using univariate logistic regression. Adjusted odds ratios and two-sided *P* values were estimated using multivariate logistic regression. Each variable was adjusted for all other variables within the table.

[ǂ]Data are expressed as mean (SD).

[a]Odds ratios were calculated per year.

[b]Odds ratios were calculated per kg/m^2.

*Two-sided *P* <0.001.

-- Data suppressed (n <10); OR and aOR not calculated.
