# Supplementary material for: Development and validation of a clinical model for preconception and early pregnancy risk prediction of gestational diabetes mellitus in nulliparous women
Source: PLoS One. 2019 Apr 12;14(4):e0215173. doi: 10.1371/journal.pone.0215173 (PMC6461273; doi:10.1371/journal.pone.0215173)
Supplement: S10 Table — (PDF) [file pone.0215173.s011.pdf]

S10 Table. Demographic and clinical characteristics of Asian nulliparous women with gestational diabetes mellitus compared to Asian nulliparous women without gestational diabetes mellitus within the California model testing subset (n=51,242) and Iowa cohort (n=323).

	California Model Testing Subset				Iowa Cohort			
	**No GDM n (%)**	**GDM n (%)**	**OR (95% CI)**	**aOR (95% CI)**	**No GDM n (%)**	**GDM n (%)**	**OR (95% CI)**	**aOR (95% CI)**
**Sample Size**	**45,087 (88.0)**	**6,155 (12.0)**			**298 (92.3)**	**25 (7.7)**		
**Age at delivery (years)**[ǂa]	29.7 (5.2)	31.7 (4.8)	1.08 (1.07, 1.09)*	1.08 (1.07, 1.09)*	30.0 (4.2)	30.3 (4.2)	1.02 (0.93, 1.12)	1.00 (0.90, 1.11)
**Expected payer for delivery**								
Government	9,582 (21.3)	928 (15.1)	0.63 (0.59, 0.68)*	0.87 (0.80, 0.94)*	15 (5.0)	--	--	--
Private	32,975 (73.1)	5,037 (81.8)	REF	REF	278 (93.3)	24 (96.0)	REF	REF
Other	2,530 (5.6)	190 (3.1)	0.49 (0.42, 0.57)*	0.56 (0.48, 0.65)*	--	--	--	--
**Smoked during pregnancy**	439 (1.0)	42 (0.7)	0.70 (0.51, 0.96)	0.74 (0.53, 1.02)	--	--	--	--
**Pre-pregnancy BMI (kg/m^2)**[ǂb]	22.1 (3.6)	23.8 (4.4)	1.11 (1.10, 1.11)*	1.11 (1.10, 1.12)*	23.5 (3.6)	24.4 (4.1)	1.06 (0.96, 1.18)	1.03 (0.92, 1.15)
**Family history of diabetes**	249 (0.6)	103 (1.7)	3.07 (2.43, 3.86)*	2.54 (1.99, 3.23)*	--	--	--	--
**PCOS diagnosis**	81 (0.2)	51 (0.8)	4.64 (3.27, 6.60)*	2.49 (1.71, 3.61)*	14 (4.7)	--	--	--
**Pre-existing hypertension**	383 (0.9)	171 (2.8)	3.34 (2.78, 4.00)*	1.68 (1.39, 2.04)*	--	--	--	--
**Pre-existing dyslipidemia**	79 (0.2)	37 (0.6)	3.45 (2.33, 5.10)*	1.85 (1.22, 2.81)	--	--	--	--
**Personal history of CVD**	41 (0.1)	--	--	--	--	--	--	--
**Assisted reproductive technology use**	500 (1.1)	134 (2.2)	1.99 (1.64, 2.41)*	1.10 (0.90, 1.35)	--	--	--	--
**Personal history of miscarriage**	107 (0.2)	24 (0.4)	1.65 (1.06, 2.57)	1.27 (0.80, 2.01)	--	--	--	--

GDM, gestational diabetes mellitus; OR, odds ratio; aOR, adjusted odds ratio; CI, confidence interval; REF, reference group; BMI, body mass index; PCOS, polycystic ovarian syndrome; CVD, cardiovascular disease

Odds ratios and two-sided *P* values were estimated using univariate logistic regression. Adjusted odds ratios and two-sided *P* values were estimated using multivariate logistic regression. Each variable was adjusted for all other variables within the table.

[ǂ]Data are expressed as mean (SD).

[a]Odds ratios were calculated per year.

[b]Odds ratios were calculated per kg/m^2.

*Two-sided *P* <0.001.

-- Data suppressed (n <10); OR and aOR not calculated.
