# Supplementary material for: Development and validation of a clinical model for preconception and early pregnancy risk prediction of gestational diabetes mellitus in nulliparous women
Source: PLoS One. 2019 Apr 12;14(4):e0215173. doi: 10.1371/journal.pone.0215173 (PMC6461273; doi:10.1371/journal.pone.0215173)
Supplement: S11 Table — (PDF) [file pone.0215173.s012.pdf]

S11 Table. Demographic and clinical characteristics of American Indian/Alaska Native nulliparous women with gestational diabetes mellitus compared to American Indian/Alaska Native nulliparous women without gestational diabetes mellitus within the California model testing subset (n=1,453) and Iowa cohort.

	California Model Testing Subset				Iowa Cohort**			
	No GDM n (%)	GDM n (%)	OR (95% CI)	aOR (95% CI)	No GDM n (%)	GDM n (%)	OR (95% CI)	aOR (95% CI)
**Sample Size**	**1,359 (93.5)**	**94 (6.5)**			--	--		
**Age at delivery (years)**[ǂa]	22.6 (5.3)	27.0 (6.5)	1.13 (1.09, 1.17)*	1.12 (1.07, 1.17)*	--	--	--	--
**Expected payer for delivery**					--	--		
Government	873 (64.2)	--	--	--	--	--	--	--
Private	431 (31.7)	48 (51.1)	REF	REF	--	--	REF	REF
Other	55 (4.1)	--	--	--	--	--	--	--
**Smoked during pregnancy**	1,213 (89.3)	--	--	--	--	--	--	--
**Pre-pregnancy BMI (kg/m^2)**[ǂb]	25.8 (5.7)	30.1 (6.5)	1.11 (1.07, 1.14)*	1.07 (1.06, 1.13)*	--	--		
**Family history of diabetes**	12 (0.9)	--	--	--	--	--	--	--
**PCOS diagnosis**	--	--	--	--	--	--	--	--
**Pre-existing hypertension**	20 (1.5)	--	--	--	--	--	--	--
**Pre-existing dyslipidemia**	--	--	--	--	--	--	--	--
**Personal history of CVD**	--	--	--	--	--	--	--	--
**Assisted reproductive technology use**	--	--	--	--	--	--	--	--
**Personal history of miscarriage**	--	--	--	--	--	--	--	--

GDM, gestational diabetes mellitus; OR, odds ratio; aOR, adjusted odds ratio; CI, confidence interval; REF, reference group; BMI, body mass index; PCOS, polycystic ovarian syndrome; CVD, cardiovascular disease

Odds ratios and two-sided *P* values were estimated using univariate logistic regression. Adjusted odds ratios and two-sided *P* values were estimated using multivariate logistic regression. Each variable was adjusted for all other variables within the table.

[ǂ]Data are expressed as mean (SD).

[a]Odds ratios were calculated per year.

[b]Odds ratios were calculated per kg/m^2.

*Two-sided *P* <0.001.

**Due to the small number of American Indian/Alaska Native women within the Iowa cohort, all data has been suppressed.

-- Data suppressed (n <10); OR and aOR not calculated.
