# Supplementary material for: Development and validation of a clinical model for preconception and early pregnancy risk prediction of gestational diabetes mellitus in nulliparous women
Source: PLoS One. 2019 Apr 12;14(4):e0215173. doi: 10.1371/journal.pone.0215173 (PMC6461273; doi:10.1371/journal.pone.0215173)
Supplement: S12 Table — (PDF) [file pone.0215173.s013.pdf]

S12 Table. Demographic and clinical characteristics of Hawaiian/Pacific Islander nulliparous women with gestational diabetes mellitus compared to Hawaiian/Pacific Islander nulliparous women without gestational diabetes mellitus within the California model testing subset (n=1,328) and Iowa cohort.

	**California Model Testing Subset**				**Iowa Cohort****			
	**No GDM n (%)**	**GDM n (%)**	**OR (95% CI)**	**aOR (95% CI)**	**No GDM n (%)**	**GDM n (%)**	**OR (95% CI)**	**aOR (95% CI)**
**Sample Size**	**1,214 (91.4)**	**114 (8.6)**			--	--		
**Age at delivery (years)**[ǂa]	24.6 (5.6)	28.2 (5.3)	1.11 (1.07, 1.14)*	1.10 (1.07, 1.14)*	--	--	--	--
**Expected payer for delivery**					--	--		
Government	601 (49.5)	--	--	--	--	--	--	--
Private	575 (47.4)	69 (60.5)	REF	REF	--	--	REF	REF
Other	38 (3.1)	--	--	--	--	--	--	--
**Smoked during pregnancy**	69 (5.7)	--	--	--	--	--	--	--
**Pre-pregnancy BMI (kg/m^2)**[ǂb]	26.9 (6.0)	29.4 (6.1)	1.07 (1.03, 1.10)*	1.07 (1.03, 1.10)*	--	--		
**Family history of diabetes**	--	--	--	--	--	--	--	--
**PCOS diagnosis**	--	--	--	--	--	--	--	--
**Pre-existing hypertension**	14 (1.2)	--	--	--	--	--	--	--
**Pre-existing dyslipidemia**	--	--	--	--	--	--	--	--
**Personal history of CVD**	--	--	--	--	--	--	--	--
**Assisted reproductive technology use**	--	--	--	--	--	--	--	--
**Personal history of miscarriage**	--	--	--	--	--	--	--	--

GDM, gestational diabetes mellitus; OR, odds ratio; aOR, adjusted odds ratio; CI, confidence interval; REF, reference group; BMI, body mass index; PCOS, polycystic ovarian syndrome; CVD, cardiovascular disease

Odds ratios and two-sided *P* values were estimated using univariate logistic regression. Adjusted odds ratios and two-sided *P* values were estimated using multivariate logistic regression. Each variable was adjusted for all other variables within the table.

[ǂ]Data are expressed as mean (SD).

[a]Odds ratios were calculated per year.

[b]Odds ratios were calculated per kg/m^2.

*Two-sided *P* <0.001.

**Due to the small number of Hawaiian/Pacific Islander women within the Iowa cohort, all data has been suppressed.

-- Data suppressed (n <10); OR and aOR not calculated.
