# Supplementary material for: Development and validation of a clinical model for preconception and early pregnancy risk prediction of gestational diabetes mellitus in nulliparous women
Source: PLoS One. 2019 Apr 12;14(4):e0215173. doi: 10.1371/journal.pone.0215173 (PMC6461273; doi:10.1371/journal.pone.0215173)
Supplement: S13 Table — (PDF) [file pone.0215173.s014.pdf]

S13 Table. Demographic and clinical characteristics of nulliparous women in other racial groups with gestational diabetes mellitus compared to nulliparous women of other racial groups without gestational diabetes mellitus within the California model testing subset (n=24,607) and Iowa cohort.

	California Model Testing Subset				Iowa Cohort**			
	**No GDM n (%)**	**GDM n (%)**	**OR (95% CI)**	**aOR (95% CI)**	**No GDM n (%)**	**GDM n (%)**	**OR (95% CI)**	**aOR (95% CI)**
**Sample Size**	**23,130 (94.0)**	**1,477 (6.0)**			--	--		
**Age at delivery (years)**[‡a]	25.5 (6.3)	29.0 (6.2)	1.09 (1.08, 1.10)*	1.09 (1.08, 1.10)*	--	--	--	--
**Expected payer for delivery**					--	--		
Government	10,475 (45.3)	521 (35.3)	0.64 (.58, 0.72)*	1.02 (0.90, 1.16)	--	--	--	--
Private	12,045 (52.1)	933 (63.2)	REF	REF	--	--	REF	REF
Other	610 (2.6)	23 (1.6)	0.49 (0.32, 0.74)*	0.63 (0.41, 0.97)	--	--	--	--
**Smoked during pregnancy**	1,015 (4.4)	66 (4.5)	1.02 (0.79, 1.32)	1.20 (0.92, 1.56)	--	--	--	--
**Pre-pregnancy BMI (kg/m^2)**[‡b]	24.8 (5.0)	27.6 (6.1)	1.09 (1.08, 1.10)*	1.09 (1.08, 1.10)*	--	--		
**Family history of diabetes**	168 (0.7)	19 (1.3)	1.78 (1.11, 2.87)	1.70 (1.03, 2.80)	--	--	--	--
**PCOS diagnosis**	46 (0.2)	17 (1.2)	5.85 (3.35, 10.23)*	3.20 (1.77, 5.81)*	--	--	--	--
**Pre-existing hypertension**	276 (1.2)	65 (4.4)	3.81 (2.89, 5.02)*	1.82 (1.36, 2.45)*	--	--	--	--
**Pre-existing dyslipidemia**	46 (0.2)	16 (1.1)	5.50 (3.11, 9.74)*	2.32 (1.23, 4.38)	--	--	--	--
**Personal history of CVD**	42 (0.2)	--	--	--	--	--	--	--
**Assisted reproductive technology use**	160 (0.7)	24 (1.6)	2.37 (1.54, 3.66)*	1.02 (0.65, 1.61)	--	--	--	--
**Personal history of miscarriage**	71 (0.3)	--	--	--	--	--	--	--

GDM, gestational diabetes mellitus; OR, odds ratio; aOR, adjusted odds ratio; CI, confidence interval; REF, reference group; BMI, body mass index; PCOS, polycystic ovarian syndrome; CVD, cardiovascular disease

Odds ratios and two-sided *P* values were estimated using univariate logistic regression. Adjusted odds ratios and two-sided *P* values were estimated using multivariate logistic regression. Each variable was adjusted for all other variables within the table.

[‡]Data are expressed as mean (SD).

[a]Odds ratios were calculated per year.

[b]Odds ratios were calculated per kg/m^2.

*Two-sided *P* <0.001.

**Due to the small number of women in other racial groups within the Iowa cohort, all data has been suppressed.

-- Data suppressed (n <10); OR and aOR not calculated.
