# Supplementary material for: Development and validation of a clinical model for preconception and early pregnancy risk prediction of gestational diabetes mellitus in nulliparous women
Source: PLoS One. 2019 Apr 12;14(4):e0215173. doi: 10.1371/journal.pone.0215173 (PMC6461273; doi:10.1371/journal.pone.0215173)
Supplement: S14 Table — (PDF) [file pone.0215173.s015.pdf]

S14 Table. **Accuracy of initial and final models among all nulliparous women in the California model testing subset and Iowa cohort.**

Model	California Model Testing Subset				Iowa Cohort			
	Number of subjects included in model	AUC (95% CI)	Calibration Plot Statistics		Number of subjects included in model	AUC (95% CI)	Calibration Plot Statistics	
			Intercept	Slope			Intercept	Slope
Initial†	352,992	0.730 (0.726, 0.733)	0.042	1.015	3,744	0.694 (0.652, 0.735)	-1.384	0.689
Final‡	353,003	0.732 (0.728, 0.735)	0.043	1.016	4,145	0.710 (0.672, 0.749)	-0.694	0.958

AUC, area under the curve; CI, confidence interval.

†Initial logistic regression model included the following variables: race/ethnicity, age at delivery, expected payer for delivery, smoking during pregnancy, pre-pregnancy BMI, family history of diabetes, polycystic ovarian syndrome diagnosis, pre-existing hypertension, pre-existing dyslipidemia, personal history of cardiovascular disease, assisted reproductive technology use, and personal history of miscarriage.

‡Final model produced after utilization of stepwise backward selection included the following variables: race/ethnicity, age at delivery (natural cubic spline transformed), pre-pregnancy BMI (natural cubic spline transformed), family history of diabetes, and pre-existing hypertension.
