# Supplementary material for: Development and validation of a clinical model for preconception and early pregnancy risk prediction of gestational diabetes mellitus in nulliparous women
Source: PLoS One. 2019 Apr 12;14(4):e0215173. doi: 10.1371/journal.pone.0215173 (PMC6461273; doi:10.1371/journal.pone.0215173)
Supplement: S15 Table — (PDF) [file pone.0215173.s016.pdf]

S15 Table. Clinical scenarios demonstrating the use of the final risk prediction model for calculating predicted risk of gestational diabetes mellitus.

**Scenario**	**Predicted risk of GDM**
Scenario 1	
Hispanic, 21 years of age at delivery, family history of diabetes	
Pre-pregnancy BMI 18 kg/m^2	3.5%
Pre-pregnancy BMI 23 kg/m^2	4.9%
Pre-pregnancy BMI 30 kg/m^2	10.3%
Scenario 2	
25 years of age at delivery, pre-pregnancy BMI 28 kg/m^2, pre-existing hypertension	
White, not Hispanic	8.6%
Hispanic	12.2%
Black	8.6%
Asian	21.5%
H/PI	15.2%
Scenario 3	
Black, 29 years of age at delivery, pre-pregnancy BMI 20 kg/m^2	
Pre-existing hypertension	4.8%
No pre-existing hypertension	2.9%

GDM, gestational diabetes mellitus; H/PI, Hawaiian/Pacific Islander; BMI, body mass index.
