# Supplementary material for: Development and validation of a clinical model for preconception and early pregnancy risk prediction of gestational diabetes mellitus in nulliparous women
Source: PLoS One. 2019 Apr 12;14(4):e0215173. doi: 10.1371/journal.pone.0215173 (PMC6461273; doi:10.1371/journal.pone.0215173)
Supplement: S16 Table — (PDF) [file pone.0215173.s017.pdf]

Table S16. Performance of different risk stratification strategies within the California model testing subset and Iowa cohort.

Model Application†	Validation Cohort	Predicted Risk Threshold	True GDM Prevalence n (%)	Women above threshold ('high risk') n (%)	Sensitivity (95% CI)	Specificity (95% CI)	PPV (95% CI)	NPV (95% CI)	Correctly Classified n (%)
All Nulliparous Women	California Model Testing Subset	3%	22,194 (6.3)	242,003 (68.6)	90.1 (89.7, 90.5)	32.9 (32.7, 33.1)	8.3 (8.2, 8.4)	98.0 (97.9, 98.1)	128,800 (36.5)
		6%		135,262 (38.3)	70.8 (70.2, 71.4)	63.9 (63.7, 64.0)	11.6 (11.4, 11.8)	97.0 (97.0, 97.1)	226,967 (64.3)
		10%		64,540 (18.3)	46.4 (45.7, 47.0)	83.6 (83.5, 83.7)	15.9 (15.7, 16.2)	95.9 (95.8, 96.0)	286.847 (81.3)
		15%		24,838 (7.0)	23.9 (23.3, 24.5)	94.1 (94.0, 94.2)	21.4 (20.8, 21.9)	94.9 (94.8, 94.9)	316,577 (89.7)
	Iowa Cohort	3%	176 (4.3)	3,441 (83.0)	94.9 (91.6, 98.1)	17.5 (16.3, 18.7)	4.9 (4.1, 5.6)	98.7 (97.9, 99.6)	862 (20.8)
		6%		1,871 (45.1)	76.7 (70.5, 83.0)	56.3 (54.7, 57.8)	7.2 (6.0, 8.4)	98.2 (97.7, 98.7)	2,368 (57.1)
		10%		939 (22.7)	48.9 (41.5, 56.3)	78.5 (77.2, 79.8)	9.2 (7.3, 11.0)	97.2 (96.6, 97.8)	3,202 (77.2)
		15%		382 (9.2)	24.4 (18.1, 30.8)	91.5 (90.6, 92.3)	11.3 (8.1, 14.4)	96.5 (95.9, 97.1)	3,673 (88.6)

GDM, gestational diabetes mellitus; CI, confidence interval; PPV, positive predictive value; NPV, negative predictive value.
†Final model included the following variables: race/ethnicity, age at delivery (natural cubic spline transformed), pre-pregnancy BMI (natural cubic spline transformed), family history of diabetes, and pre-existing hypertension.
